# Reconstructing Coherent Networks from Electroencephalography and Magnetoencephalography with Reduced Contamination from Volume Conduction or Magnetic Field Spread

**DOI:** 10.1371/journal.pone.0081553

**Published:** 2013-12-02

**Authors:** Mark Drakesmith, Wael El-Deredy, Stephen Welbourne

**Affiliations:** 1 School of Psychological Sciences, University of Manchester, Manchester, United Kingdom; 2 Cardiff University Brain Imaging Research Centre (CUBRIC), Cardiff University, Cardiff, United Kingdom; University College of London - Institute of Neurology, United Kingdom

## Abstract

Volume conduction (VC) and magnetic field spread (MFS) induce spurious correlations between EEG/MEG sensors, such that the estimation of functional networks from scalp recordings is inaccurate. Imaginary coherency [1] reduces VC/MFS artefacts between sensors by assuming that instantaneous interactions are caused predominantly by VC/MFS and do not contribute to the imaginary part of the cross-spectral densities (CSDs). We propose an adaptation of the dynamic imaging of coherent sources (DICS) [2] - a method for reconstructing the CSDs between sources, and subsequently inferring functional connectivity based on coherences between those sources. Firstly, we reformulate the principle of imaginary coherency by performing an eigenvector decomposition of the imaginary part of the CSD to estimate the power that only contributes to the non-zero phase-lagged (NZPL) interactions. Secondly, we construct an NZPL-optimised spatial filter with two *a priori* assumptions: (1) that only NZPL interactions exist at the source level and (2) the NZPL CSD at the sensor level is a good approximation of the projected source NZPL CSDs. We compare the performance of the NZPL method to the standard method by reconstructing a coherent network from simulated EEG/MEG recordings. We demonstrate that, as long as there are phase differences between the sources, the NZPL method reliably detects the underlying networks from EEG and MEG. We show that the method is also robust to very small phase lags, noise from phase jitter, and is less sensitive to regularisation parameters. The method is applied to a human dataset to infer parts of a coherent network underpinning face recognition.

## Introduction

As cognitive function arises from the dynamic interaction between brain regions, there is an increasing interest in moving cognitive brain imaging beyond the identification of anatomical loci of functional processes, to the detection of the underlying functionally connected networks. Functional relationships between brain regions can be inferred from correlated haemodynamic or electrophysiological signals. The shortcomings of using the haemodynamic response as the basis of detecting correlated regions due to poor temporal resolution have been documented [3]. The millisecond temporal resolution of electroencephalography (EEG) and magnetoencephalography (MEG) should make them ideal tools to measure functional connectivity, since the time scales of neural interaction are also of this order. However, two challenges arise from using scalp recording to infer interacting networks: Volume conduction (VC) and magnetic field spread (MFS) cause smearing of the effect of the neural generators at the surface and result in poor spatial resolution. Further, VC and MFS introduce spurious and erroneous correlation in the recorded signals such that the estimated networks are inaccurate. Here we propose a physiologically valid method to reduce the effects of VC and MFS in estimating coherent source networks. Before introducing the proposed method, we review coherence as a method of inferring functional connectivity and dynamic imaging of coherent sources (DICS) [2] as a method of estimating coherence between reconstructed sources. The issues of VC and MFS are discussed further along with the obstacles they present to uncovering true source level coherences, as well as previous attempts to solve the problem.

### Beamformers as a Solution to the Inverse Problem

Overcoming the poor spatial resolution of EEG/MEG has been the focus of a great deal of research (see [4], [5] for reviews). This generally involves the calculation of a linear forward solution, or lead field, which describes the transformation of the signal from the neural generators to the surface sensors. This is followed by an attempt to reverse the calculation to solve its inverse. The inverse solution is problematic as it attempts to describe a complex dynamical system from a relatively small number of observations. The inverse problem is described as ‘ill-posed’ as it has no unique solution [6]. Solutions such as the linearly constrained minimum variance (LCMV) beamformer [7] do not explicitly try to solve the inverse problem, unlike dipole fitting methods [8], but instead rely on spatial filters that weight the estimated sources as a function of the covariance matrix of the time-series. A spatial filter is constructed for each source point (voxel) such that the variance of the total source power is minimal while keeping the output of the filtered lead field constant. This maximises the beamformer output for the target source while contributions from other sources are attenuated (however signals from strong, nearby sources may subdue output from the target source). Beamformers have been shown to be a useful method for identifying EEG/MEG sources (see [9] for a review). However, the fact that beamformers assume that distinct sources are uncorrelated may pose problems when trying to infer functional connectivity between reconstructed sources. This issue will be addressed in more detail in the discussion.

Another issue concerning beamformer methods is that of regularisation. The spatial filter requires regularisation to prevent overfitting. Higher regularisation is preferred to reduce the chance of false positives, but this results in the smoothing of sources. Lower regularisation allows for more focal sources to be reconstructed, but is more prone to false positives [10], [11]. This is described mathematically in the theory section.

A common application of the beamformer technique is to estimate the source-level time-series. This type of approach is often referred to as “virtual electrode” methods, because they can be conceptualised as placing virtual electrodes into voxels. The most obvious method of investigating functional connectivity at the source level would be to estimate time-series for pairs of voxels and measure the functional connectivity between them. While superficially very attractive, this approach needs to be treated with some caution when used in the context of functional connectivity estimation, because uncertainties in the reliability of the reconstructions can give rise to systematic errors, which will contaminate subsequent connectivity estimates [12]. Our preference is to use DICS, a modified beamformer method (described later), which allows for coherence based source connectivity estimates to be computed in a single step.

### Coherence

Coherence is a statistic that often increases when activity in neural assemblies is functionally synchronised and as such can be taken as a measure of functional connectivity in electrophysiological data (see [13] for a review). Coherence is thought to be the mechanism by which percepts are bound together: The “binding hypothesis” [14], [15]. Electrophysiological studies recording multi-unit activity and local field potentials have illustrated that coherence accurately reflects both intra-cortical and inter-cortical communication (see [16]–[18], for reviews).

In practice, coherence describes how closely related the spectral densities of two signals are, and so is equivalent to a correlation coefficient in the frequency domain. Coherence is the absolute value of coherency, the complex-valued ratio between the cross-spectral density (CSD) and the individual auto-spectra (or power) of two signals, i.e. a covariance matrix in the frequency domain. This can be calculated by Fourier transformation or wavelet convolution of the cross-covariance of the two time-series [19]. Since the ratio between the individual auto-spectra and the cross-spectra are complex, they embody both correlation of power amplitude and phase synchrony of the signals.

While coherence analysis is simple and physiologically-valid, the interpretation of its spatial characteristics at the sensor level is difficult, due to the irregular way in which the source activity manifests. This provides a strong motivation for looking at connectivity at the source-level. Using the ‘virtual-electrode’ method described above is an obvious means of achieving this. This however is computationally demanding and can suffer a number of pitfalls detailed in the sections below.

### Dynamic Imaging of Coherent Sources

Dynamic imaging of coherent sources (DICS) [2] is a beamformer technique that operates under the same principle as LCMV, but with two principle differences: (1) The covariance matrix in the calculation of the spatial filter is replaced by the sensor-level CSD matrix. (2) The filter is applied to the sensor-level CSD to reconstruct the source-level CSDs of all combinations of pairwise voxels. From these the source-level coherences between sources can be estimated. Thus DICS differs from other beamforming methods in that it directly estimates the interactions between sources as well as their individual powers.

DICS is advantageous over separate source-reconstruction and functional connectivity combinations because functional interactions are reconstructed as source pairs. The assumptions underlying the localisation and the estimation of coherence of source pairs are the same. This reduces the confounds of systematic bias on the coherence estimates. Another significant benefit is the massive reduction in computational demands. The prospect of whole-brain source-level connectivity inference is more computationally tractable when the step of reconstructing the source-level time-series is bypassed.

A full description of DICS is given in [2], [10]. DICS has been applied to MEG data to investigate coherence in a number of cognitive phenomena such as reading [20], motor control [21] and binocular rivalry [22]. For EEG, there are some recent examples of applying DICS to EEG data [23], [24] although these did not reconstruct cortico-cortical coherence, but rather sources coherent with an external EMG signal. To our knowledge, only one study has successfully inferred cortico-cortical network using DICS with EEG recordings [25]. They implemented a variation of the standard DICS method where the real-valued spatial filter in the direction of maximum variance was used to estimate source CSDs. Generally, VC limits the applicability of DICS to EEG as detailed below. In it’s current form, DICS has no means of reducing artefactual connectivity that arises due to VC. The present study is the first to use DICS to infer cortico-cortical connections using EEG, by explicitly minimising the effects of VC.

### Volume Conduction (VC) and Magnetic Field Spread (MFS)

Source reconstruction is generally poorer for EEG than MEG due to the low conductivity of the skull, which leads to attenuation and spatial smearing of source currents. A more specific confound exists with regards to coherence, in that the brain tissue, being highly conductive in comparison to the skull, can result in currents being conducted to distal electrodes [26], [27]. The current flow is highly dependant on anatomical factors, including structural discontinuities in the skull, lesions [28], and anisotropic conductivity of white matter [29]. Although these currents can be modelled during source analysis, EEG sensors are generally maximally sensitive to the region directly beneath the electrode. Therefore, additional contribution to the EEG signal from distal sources will significantly impact on the mapping of these signals to source space. The calculation of the lead field requires precise tissue segmentation and assigning accurate conductivity values to each compartment, using boundary element method (BEM) or more recently, finite element method (FEM) models (see [30] for a review). This introduces several levels of potential errors that can have very large effects. For example, even very small holes (less than 1 mm) in the skull can drastically alter the flow of volume currents [31], resulting in inaccurate localisation of sources as well as false positives when reconstructing source-level coherence.

MEG is generally considered less susceptible to VC [27], [28], [32]. Secondary volume currents can theoretically induce equivalent magnetic fields confounding the primary neuromagnetic fields; however, these effects are negligible in comparison to EEG [33]. Head tissues are permeable to magnetic fields, so the neuromagnetic fields are less dependent on anatomy than EEG. However, due to the effects of magnetic field spread (MFS), artefacts can still arise. These artefacts are maximal over short distances but can extend over large areas of the topographic surface, and therefore can manifest even at long-range connections. Unlike VC, however, MFS is not dependent on specific tissue conductivities. Source localisation can theoretically be attained by simple inversion of the Biot-Savart law. A volume conductor model is still necessary to account for the impressed currents of a dipole [34].

A basic model-free method of overcoming the problem of VC in EEG in determining surface coherences is to take the Laplacian of the surface potentials [35], [36]. This acts as a high-pass spatial filter, emphasizing sources at smaller spatial scales. This method is heavily dependent on the spatial distances between coherent sources. As such, valid, short-range coherences may be removed while erroneous, long-range volume currents may be retained. Imaginary coherency (described below) offers an improvement on this providing a non-spatially dependent way of removing VC.

### Imaginary Coherency

Nolte *et al* [1] proposed the concept of imaginary coherency as a model-free way of dealing with VC artefacts when calculating coherence at the sensor level (this is detailed in the theory section below). It is based on two critical assumptions: Firstly, that true neural interactions must have some phase lag and, secondly, that VC coherences are always instantaneous with zero phase lag. The first assumption can be justified in that even when studies describe phase lags between populations as instantaneous, there is still a phase lag of a few milliseconds or even microseconds [37]. There may be chance instances of phase difference too small to give a meaningful imaginary component, but if the CSD is calculated for a sufficiently long time-series, or is averaged over a sufficient number of event-related epochs, this effect will be negligible. There is also the possibility of sustained reciprocal interactions where there is zero phase lag and the phase lag does not vary at all over time. Imaginary coherence would not be able to detect this type of interaction (this issue is elaborated on further in the discussion).

The second assumption is valid given that VC can be described by the quasi-static approximation of Maxwell's equations [38]. The approximation describes the dynamics of VC without time-derivatives: They are treated as effectively instantaneous and hence do not contribute any phase lag. There is experimental evidence that the approximation is justified for frequencies below 1 kHz [39], which is well within the frequency range typically analysed in human EEG/MEG.

Nolte *et al* [1] successfully used imaginary coherency to identify interhemispheric coherence between electrodes on the motor regions during finger movement. Imaginary coherency has been used in a number of other EEG studies. For example, [40] correlated imaginary coherency with certain phases of brain maturation.

A few recent studies have attempted to utilise this to uncover phase-lagged coherences at the source level. A recent method for examining source dynamics with imaginary coherency is that of Marzetti *et al* [41]. Here, the imaginary CSD is approximated by a model in a modified principle component analysis (PCA) technique to separate contributions of interacting sources. This is a qualitatively different approach to beamformer, as it does not scan each source (or source pair) independently for their contribution to the sensor data. Other recent studies [42]–[44] have adopted a two-step approach to the problem, by estimating source level time-series using the virtual electrode method and then computing imaginary coherency between the estimated source-level time-series. This approach reduces spurious interactions that arises from smoothing of the source space inherent in source localisation algorithms. This approach suffers the same limitations as other connectivity methods based on reconstructed source time-series. It also requires additional processing time due to the separate generation of source time-series and coherence calculations. This makes it unfeasible to do whole-brain network analysis [45] As a result *a priori* selection of regions of interest (ROIs) has to be performed which can be subjective. In addition to these drawbacks, VC/MFS artefacts at the sensor-level, if not accurately modelled, can lead to source mislocalisations [29], [32]. While computing imaginary coherency from source-level time-series will reduce artefactual connectivity estimates, it can not resolve any effects VC/MFS may be projected to the source space. To reduce the likelihood of these errors, it would be more prudent to remove VC/MFS artefacts at the sensor-level prior to source reconstruction.

Another approach to overcoming VC/MFS at the sensor-level is the phase lag index (PLI) [46]. This attempts to overcome the issue with imaginary coherency that it is sensitive to the size of the phase lag as well as the strength of coupling. For example, if two interacting pairs of sources have equal coupling strength but one has a larger phase difference than the other, imaginary coherency will be biased towards the connection with the larger phase lag. PLI overcomes this issue by measuring the asymmetry of the distribution of phase lags, as symmetrical phase lag distributions are more likely to arise where the phase lag is at 0 or π. As a result, this measure is less sensitive to the degree of phase lag, only the presence of phase lag. So far, one study has extended this principle to infer connectivity between virtual electrode time-series [47].

### Aims

This study proposes an adaptation of the DICS method, in order to reduce the effect of VC and MFS artefacts in reconstructing source-level coherent networks, and therefore improve recovery of source-level coherences. In contrast to the methods cited in the section above, this will allow source-level CSDs to be computed directly, making the process more computationally efficient and negating the potential pitfalls of a separate stage of reconstructing the source-level time series. Our approach is two-fold. First, we reformulate the approach of imaginary coherency to increase its sensitivity, by constructing a non-zero phase-lagged (NZPL) CSD matrix. Secondly, we use the same approach of DICS to solve a problem analogous to the standard M/EEG inverse problem, with an additional *a priori* assumption that true sources have non-zero phase lags.

The theory for the NZPL CSD and its application to DICS is presented first, followed by the results from experimental simulations comparing network reconstruction using DICS with the full CSD to that using the NZPL CSD. Simulations were carried out to comparing the performance of these two methods at different levels of phase jitter, phase lags and regularisation parameters. Finally, the applicability of the method is illustrated on a set of real EEG recordings.

## Theory

### Source Reconstruction

All reconstruction methods estimate the solution to the inverse problem:



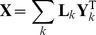
(1)


The lead field, **L** *_k_*, is an *n_s_*×3 matrix, which describes the contribution of source *k* to the sensors in 3 directions in Cartesian space where *n_s_* is the number of sensors. **X** is the sensor data in the time or frequency domain. The beamformer method estimates a solution to the inverse problem by using a spatial filter **W** *_k_* to create an estimate of the source activity 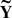.



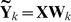
(2)


In the LCMV and DICS beamformers, **W** *_k_* is estimated by solving the constrained minimisation problem:



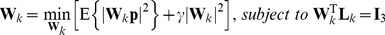
(3)


Where E denotes the expected value, **p** is the Fourier transformed data and *γ* is the Tikhonov regularisation parameter.

The solution to this is given by [7]:



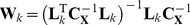
(4)


For DICS, the spatial filter for two source points are multiplied with the sensor level CSD (sCSD), **C_X_** to create a 3×3 matrix of reconstructed source CSDs (rCSD) between the 3 Cartesian components of sources *k* and *l*.



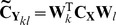
(5)


The highest singular value of this matrix is treated as the amplitude of the rCSD between *k* and *l* [2] when this is significantly larger than the next singular value. Otherwise, the trace of the matrix is taken. The spatial filter accommodates Tikhonov regularisation by substituting **C_X_** for:



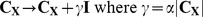
(6)


Where *γ* is the absolute regularisation parameter, which is a multiple of the Euclidian norm of the sCSD matrix, while *α* is the relative regularisation [10]. This parameter prevents over-fitting in a minimum norm solution to an ill-posed problem. It adjusts the sensitivity of the spatial filter according to the expected distribution of sources. More focal sources are favoured by low regularisation, but risks introducing false positives, while sources that are more diffuse are favoured by high regularisation, but risks making false negatives. Larger *α,* therefore reduces the acuity of the spatial filter, so given data of sufficient quality, lower regularisation parameters are preferred. Until recently there was no robust benchmark for choosing an appropriate regularisation for spatial filtering. However, there is a recent method for estimating the optimal regularisation parameter from the condition number of the matrix [11].

### Imaginary Coherency

This section describes the motivation for using the imaginary coherency [1] to quantify true neural interactions from sensor-level time-series.

The Fourier transformed sensor time-series *p* from sensor *i*, is given by:



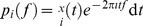
(7)


For simplicity and clarity, the notation *f* is removed from here on; all variables are still functions of frequency. *ι* denotes the imaginary unit. As per the convolution theorem, the cross-spectra can be calculated directly from the complex conjugate product of the two Fourier transformed signals. These are averaged across a number, *n_e_*, of epochs to estimate the true cross-spectral density (CSD) matrix.



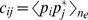
(8)


The complex coherency function between two signals *i* and *j* is the ratio of the cross-spectra to the auto-spectra:



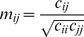
(9)


The absolute value of which is usually taken as a measure of coherence, while the imaginary part is a measure of the phase-lagged coherence. The Fourier transformed data can be expressed in polar form:



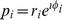
(10)


Where *r_i_* is the amplitude and *φ_i_* is the phase, the CSD for a single sample can be expressed as:



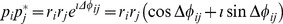
(11)


Where 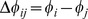 is the phase lag between signals *i* and *j*. When there is no phase lag (Δ*φ* = 0), cos Δ*φ* = 1 while sin Δ*φ* = 0 and when the lag is maximum (Δ*φ* = π/2), cos Δ*φ* = 0, while sin Δ*φ* = 1. When averaged across samples, the amplitudes *r_i_r_j_* will also affect the imaginary coherency (unless the amplitude is constant across all trials, which is unlikely).

The real and imaginary parts of coherency can, in this case, be treated as representing the proportion of the CSD with zero phase lag and that with maximal phase difference (π/2). Independent signals will lead to random *φ_i_* but also small *r_i_* so these will not contribute significantly to imaginary coherency. As VC/MFS coherence is only instantaneous, the removal of the real part removes the contribution of VC/MFS (at least the first order effects) to the connectivity inferred from the coherence calculations.

### Non-Zero Phase-Lagged (NZPL) CSD and Coherence

In this section we reformulate the principle of imaginary coherency to improve its sensitivity to non-zero phase lags.

While instantaneous and phase-lagged components can be easily separated in the off-diagonal elements, the contribution of these two components to the full power is still unknown. The existing imaginary coherency approach does not take into account the dependence of phase lag on the imaginary CSD. This results in a biased estimate of non-instantaneous interactions (see appendix S1). We therefore wish to identify the components in the power that contribute only to the imaginary part of the CSD. To demonstrate this, we consider the eigenvector decomposition of the full CSD, for each sample to give the full power, **p**.

The CSD calculation in equation 8 can be expressed as an outer product of two vectors, averaged across samples:



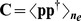
(12)


The decomposition of a single sample is given by:



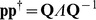
(13)


Where **Q** = [**q**_1_…**q** *_ns_*] is a matrix of column eigenvectors and **Λ** = [*λ*_1_…*λ_ns_*]^D^ is a diagonal matrix of corresponding eigenvalues. This decomposition yields one non-zero eigenvector/eigenvalue, which satisfies:



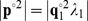
(14)


The superscript ^°2^ represents the Hadamard (element-wise) square function. We can obtain an equivalent representation of the power contributing only to the imaginary part of the CSD by performing the same decomposition on the imaginary CSD:

Any matrix can be uniquely decomposed into one symmetric (or Hermetian) and one anti-symmetric (or anti-Hermetian) matrix. In this case, these equate to the real and imaginary parts, respectively. These two matrices can then be decomposed further into two sets of eigenvectors/eigenvalues.



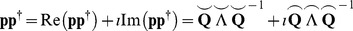
(15)


In the case of the symmetric real part, there are two non-zero eigenvector/eigenvalues (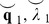
and 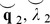), which represent the instantaneous contributions to the power. In the case of the anti-symmetric imaginary part, there are two non-zero eigenvector/eigenvalues. These eigenvectors are purely imaginary and exist as a complex conjugate pair (i.e. 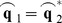 and 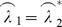). The eigenvectors yielded by the real decomposition relate to the full power in a similar way to equation 14, by satisfying:



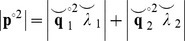
(16)


A term representing the power that arises due to the phase-lagged interactions can therefore be obtained by using the analogous terms from the imaginary decomposition (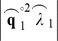). By populating the diagonal of the imaginary CSD with this term, we obtain:



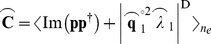
(17)


Where the superscript ^D^ indicates a vector expressed as a diagonal matrix. In this version of the CSD, a more accurate estimate of the auto-spectra contributing to the non-instantaneous interaction within that sample is used. The NZPL coherence can then be computed in the same way as standard coherence:



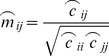
(18)


This results in a more sensitive measure of non-instantaneous interactions and is less susceptible to bias from phase lag. This approach as the same advantage as the PLI method [46], however, by manipulating the CSD directly, the NZPL approach can be extended to source localisation directly, without the need to reconstruct virtual electrodes [47] (see below).

### Higher-Order Artefacts

**Figure 1:**
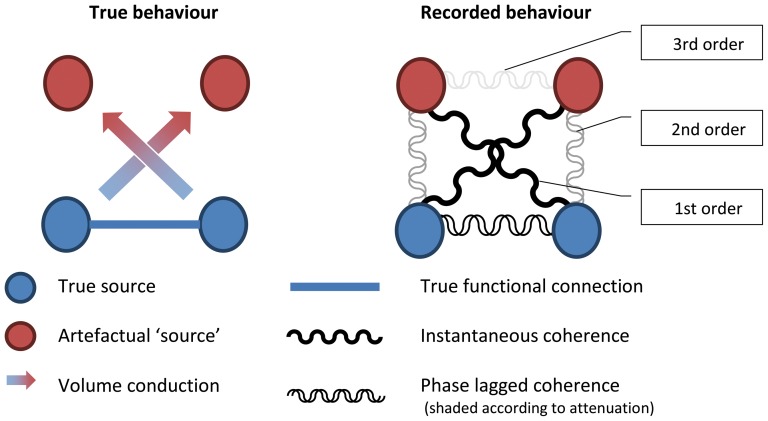
An illustration of the effects of higher order artefacts. Contralateral 1st order artefacts are instantaneous, but there are smaller 2nd order artefacts from the true sources and the ipsilateral VC ‘source’ that are phase-lagged. In addition, there are also much weaker 3rd order artefacts between the VC ‘sources’. This is a simplified representation of the coherence patterns reported in supplementary material.

It should be noted that imaginary coherency, PLI and the NZPL method are restricted to removing first order artefacts, that is artefacts that arise due to spurious interactions between the true signal and its VC ‘echo’. While this interaction will be instantaneous, if there is a true (phase-lagged) interaction with a second source, this will inevitably be phase-lagged with respect to the VC echo of the first source and, therefore, produce a phase-lagged coherence. We will refer to this interaction as a second order artefact. In addition, if a VC echo of the second source arises, then there will also be phase-lagged coherence with the first VC echo. This we call a third-order artefact. The issue is represented graphically in figure 1.

While the NZPL CSD in itself does not deal with this issue, its application to spatial filtering can minimise the effects of higher order artefacts. Assuming a VC echo is always smaller in magnitude than the originating signal, the higher-order interactions will likely be small enough to be adequately suppressed by the correlation minimisation constraint of the spatial filter. This approach is detailed in the section below.

It should be noted that while an imaginary component in the CSD in sensor space implies a phase-lagged interaction in source space, the absence of the imaginary component in sensor space does not necessarily imply the lack of a phase-lagged interaction in source space. Therefore there are many potential source interactions that will be invisible to the sensors. This issue is inherited from the general ill-posed nature of the EEG/MEG inverse problem.

### Reconstructing NZPL Sources

Having established a method to more accurately identify non-instantaneous interactions, we apply the new NZPL CSD matrix to the problem of reconstructing coherent sources. We impose additional assumptions to that of the standard DICS approach (see [48]–[52] for similar approaches). Using the *a priori* assumption that truly interacting sources will always have non-zero phase lag, we create a representation of these signals as projected to the sensor level. To estimate this we need representations of both the auto-spectra and cross-spectra of these signals.

The projection of the full CSD from sources to sensors can be expressed as:



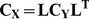
(19)


The imaginary part of the sCSD can be obtained using the same projection.



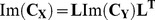
(20)


The NZPL CSD at the source level can also be projected in the same way.



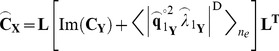
(21)


The projection of the imaginary cross-spectra of the formula can be derived easily from the imaginary sCSD.



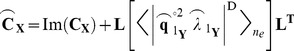
(22)


However, the NZPL auto-spectra part of the equation cannot be easily derived from the sensor data as it requires *a priori* knowledge of the separation of instantaneous cross-talk from the real part of the phase-lagged interaction. We therefore make a second assumption that the NZPL operation applied to the sCSD will sufficiently model the projection of the NZPL source power to the sensor level.



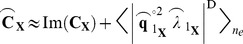
(23)


This formulation comes at the expense of omitting the cross-talk that inevitably arises in this projection of the auto-spectra. Our assumption is that there is sufficient information about this cross-talk in the higher-order artefacts (see above) that remain in the imaginary part of the sCSD.

This estimate of the projected source NZPL CSD is then used to compute a spatial filter optimised to recover NZPL interactions, using the same covariance minimisation constraint as per the existing DICS method.



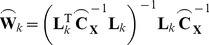
(24)


The use of the NZPL sCSD in the spatial filter calculation provides a more optimal reconstruction of the rCSD than the standard spatial filter. The NZPL filter selectively supresses signals that do not arise from non-instantaneous interacting sources, compared to the standard filter, which treats instantaneous and non-instantaneous interactions equally. This results in more signal from non-instantaneous interacting sources being attributed to the target sources (essentially treating a pair of phase-lagged interacting sources as a single source). This has two advantages: (1) It will lead to a beneficial overestimation of the non-instantaneous interactions when projecting sensor data to source space. (2) It will lead to an increase in the apparent SNR of the signal, resulting in greater spatial acuity of the filter [11]. The NZPL filter is still able to supress the higher-order artefacts that remain present in the imaginary part of the sCSD. These artefacts are much weaker than the true instantaneous interactions and therefore more readily suppressed by the filter. Appendix S2 uses a simple simulation, based on more straightforward mapping between sensors and sources, to illustrate the properties and benefits of the NZPL spatial filter compared to the standard filter.

Once the NZPL spatial filter is constructed, it is straightforward to project the NZPL sCSD to source space in the same way as DICS (as per equation 5):



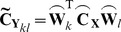
(25)


This creates an rCSD where non-instantaneous interactions are overestimated. 1^st^ order artefacts are removed completely by the NZPL manipulation while higher-order artefacts are reduced by the minimisation constraint of the spatial filter. This approach significantly reduces the projection of artefactual VC/MFS interactions into the rCSD, improving the accuracy of the inferred connectivity.

## Experiments

### General Method

A simulation of a simple coherent network was used to test the performance of the NZPL DICS method. From this, the forward solution was used to calculate simulated EEG and MEG recordings. These were used to reconstruct a source-level coherent network using an sCSD calculated in the standard way (full sCSD) or the NZPL sCSD. A number of experimental manipulations were carried out to test the effects of phase-jitter, phase lag and regularisation on the performance of DICS in each case. A quantification of the effect of phase lag and noise on coherence computed with NZPL CSDs is also made.

### Network Simulation

**Figure 2:**
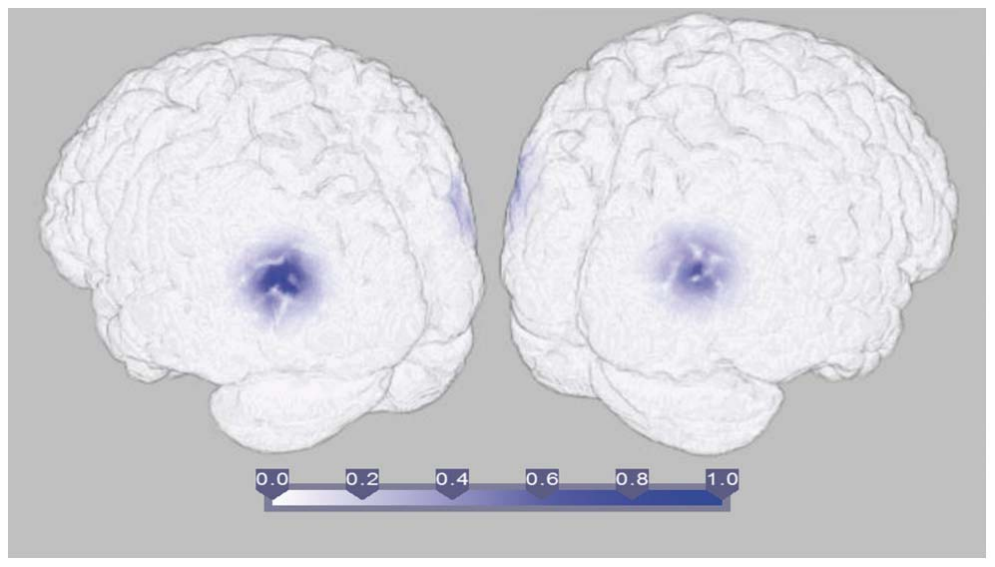
Positions of sources used in all simulations. MNI coordinates: [−40.5 −72.5 17] and [42 −72.5 17]. Colour bar indicates normalised source strength.

Neural activity was simulated in a regular 7.5 mm^3^ grid of 1454 dipoles restricted to superficial grey matter in a standard MNI T1-weighted MRI template. All dipoles were orientated orthogonally to the head surface. All dipole time-series were simulated with Gaussian white noise to simulate the presence of non-correlated activity. Two cortical regions in opposite occipital lobes were selected as nodes for the coherent network (MNI coordinates: [−40.5 −72.5 17] and [42 −72.5 17] separated by a distance of 82.5 mm). One voxel was selected as the centre of the node and activity in that node was smoothed to surrounding voxels with a FWHM of 5 mm^3^ (see figure 2).

Equal pre- and post- stimulus periods of 1000 ms were used. In the post-stimulus period, activity in the two occipital nodes was simulated using a 33 Hz sinusoidal wave. The two sources were synchronised with a jittered phase lag sampled from a von Mises distribution (a Gaussian distribution across a circle) with mean of 0.5π radians and a FWHM of 0.25π radians. In the pre stimulus period, noise was generated from the same oscillatory activity used in the post-stimulus period, but with a completely random phase lag sampled from a uniform circular distribution. This ensured the pre- and post- stimulus periods has the same power in the frequency band of interest. Additional Gaussian noise was superimposed on the signal with a signal-to-noise proportion (SNP) of 0.9, equivalent to an SNR of 9.54 dB, which is in the range of SNRs typically seen in evoked EEG responses [53]. The amplitude of the total signal was constant at 1 nA. These simulations were repeated over 100 epochs.

### Forward Calculations

Lead fields for both EEG and MEG were calculated using FieldTrip [54]. The EEG lead field was based on a VC model created using the boundary element method (BEM) [30]. The MNI template brain was segmented into brain, skull and scalp compartments defining 3 homogeneous conductive mediums with conductivity values of 33 mS/m, 0.41 mS/m and 33 mS/m, respectively. The scalp potentials were calculated using the forward solution (equation 1) and sampled by 64 scalp electrodes based on a Biosemi64 scalp electrode array with the reference electrode placed at infinity.

The MEG lead field was based on a single-shell model from a grey-matter segmentation of the same MRI template brain. The lead field was calculated using the quasi-static approximation of the magnetic forward model, described by the Biot–Savart law [34]. The forward solution was used to calculate simulated readings for 148 axial gradiometer sensors based on the configuration of a 4D MAGNES 2500 WH scanner. The sensor data for both modalities was obtained from the linear forward solution in equation 1. Example forward solutions for one simulation are shown in appendix S3.

### Sensor-Level Cross Spectral Density (sCSD)

The Fast Fourier transform was applied to the full length of the time-series to obtain the complex spectra for each channel. The full sCSD, and the NZPL sCSD were calculated as per equations 12 and 17, respectively. The sCSD was averaged over the frequency band of interest (25–40 Hz) and across the 100 epochs.

### Source Coherences

DICS [2] was performed to reconstruct source coherence across all voxel combinations, using the same source positions defined in the source simulations (1454 sources restricted to superficial grey matter). Spatial filters were calculated using either the full, or NZPL sCSDs, as per equations 4 and 24, respectively. A relative regularisation parameter of α = 10^−6^ was used. The rCSDs corresponding to the two sCSD types were then estimated using equations 5 and 25, respectively. Source-level coherences for both rCSD types were calculated in the standard way using equation 9 for the pre and post stimulus matrices. Systematic bias and filter leakage was removed by subtracting the pre-stimulus reconstruction from the post-stimulus reconstruction. This noise-contrasted matrix was used as the measure of reconstructed source-level coherences.

### Performance Measurement

The performance of the whole-brain network reconstruction was evaluated by comparing the “true” matrix with the reconstructed matrix across a range of thresholds. The “true” matrix consisted of the signal from the active source pair smoothed out across the connection space using a Gaussian kernel with a FWHM of 5 mm^3^. The Gaussian-smoothed network deals with spatial inaccuracies across a gradient of distance by progressively penalising reconstruction performance as it moves further away from the centre of true activity. The noise-contrasted reconstructed network was thresholded at 120 equally spaced values from the lowest to the highest values in the matrix. The true positive rate (TPR) and false positive rate (FPR) were calculated from the proportions of all sources, which are identified as true or false at the given threshold. The TPR and log FPR provided the points for the log receiver-operator characteristic (ROC) curve. The log ROC curve was used in preference to a standard ROC curve because of the large number of negative values. This was proposed as a method of scaling the ROC curve where there is a large ratio of negatives to positives, as there is here [55]. Finally, the area under the log ROC curve (AUC) was calculated by integrating the log ROC curve using the trapezium rule. The AUC measure gives a single value that quantifies the accuracy of each reconstruction. 10 runs of each experiment were carried out to provide a measure of variance for these AUC values.

### Statistical Analysis

Log ROC AUCs for each condition were tested for significant increase from a critical AUC value using one-tailed single sample *t*-tests. The critical AUC was defined as the log ROC AUC computed for an overlap between the FPR and TPR distributions of *p* = 0.05 (with the assumption that the FPR and TPR distributions are Gaussian). The critical AUC is approximately 3.84. Paired sample *t*-tests were also performed to test for significant increases in AUCs for the reconstructions with the NZPL sCSD compared to the reconstructions for the full sCSD. Bonferroni correction was applied to all *t*-tests.

### Reconstruction Performance and Effects of Phase Jitter

#### Method

To assess the effects of phase jitter (i.e. the FWHM of the phase lag distribution) the simulations were repeated where the phase jitter between the two oscillatory sources was varied. FWHMs of 0, π/32, π/16, π/8, π/4, π/2 and π radians were tested.

#### Results

**Figure 3:**
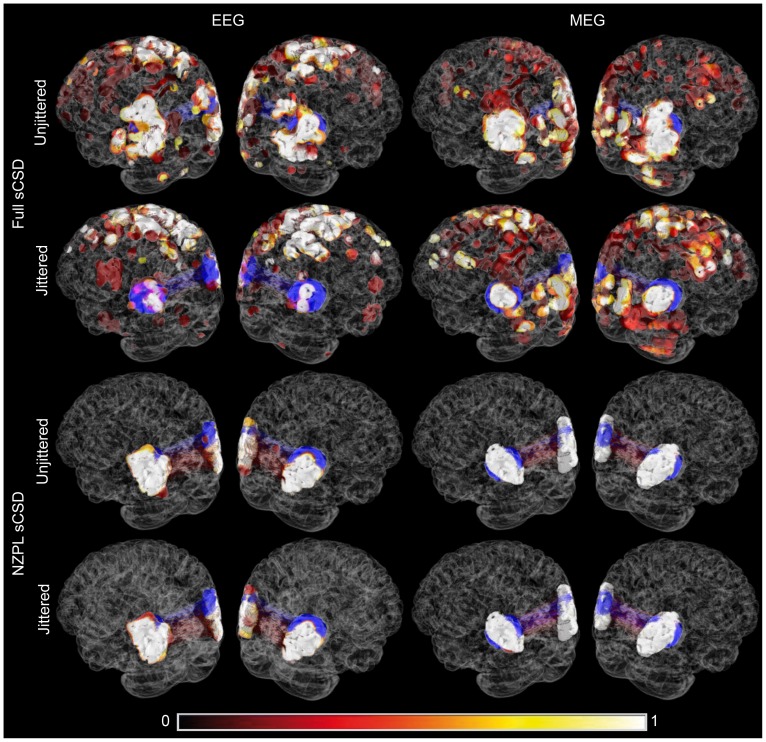
Reconstructed network with phase jitter FWHM of 0 and π/4 calculated from full and NZPL sCSDs for EEG and MEG. Colour code indicates the proportion of trials in which a voxel pair lies in the top 0.01% of the coherence matrix. Blue indicates location of the original network nodes.

**Figure 4:**
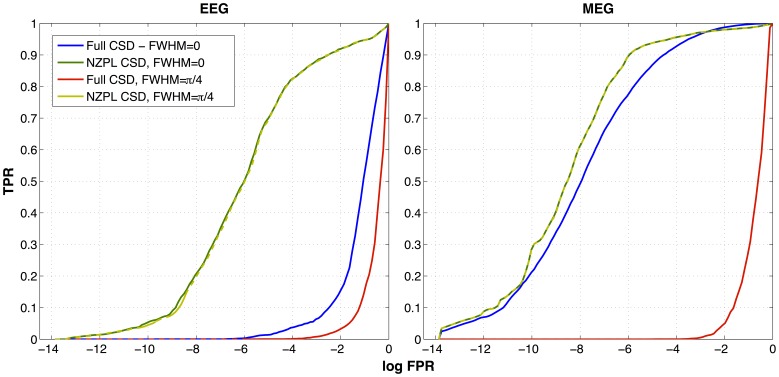
Log ROC curves for performance of the DICS reconstruction for noise-normalised coherences for EEG and MEG reconstructions, plotted for full and NZPL sCSDs, for zero and non-zero (π/4) phase jitters. FPR =  false positive rate, TPR =  true positive rate.

Examples of reconstructed networks for unjittered and jittered (FWHM = π/4) conditions are shown in figure 3 for each reconstruction method and imaging modality. The corresponding log ROC curves are shown in figure 4. For the EEG full sCSD, there is very poor reconstruction of the original network in both jittered and unjittered conditions. There was improvement when using the NZPL sCSDs, which appears to provide good reconstruction of the original network in both conditions. For MEG, the full sCSD without jitter partially reconstructs the correct network but with some false positives. The jittered conditions show poor performance as with EEG. In both cases, the NZPL CSD provides a very accurate reconstruction.

**Figure 5:**
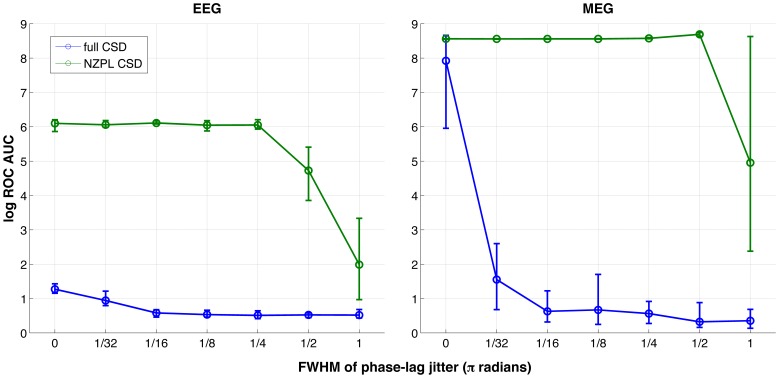
Log ROC AUCs calculated for noise-normalised coherence for EEG and MEG reconstructions, across phase jitter FWHMs.

The AUCs for reconstructions across all jitter FWHMs are shown in figure 5 and summarised in table 1. Single sample *t*-tests show that in EEG and MEG, all reconstructions using the full sCSD were not significantly above the critical AUC, with the exception of the unjittered MEG reconstruction (*t*_(9)_ = 17.74, *p*<10^−7^), indicating DICS with the full sCSD is very intolerant of phase jitter. In contrast, the AUCs for reconstructions using the NZPL sCSD were significantly above the critical AUC across all jitter FWHMs, with the exception of the largest FWHM (π radians), where EEG reconstruction was non-significant and MEG reconstruction was borderline significant (*t*_(9)_ = 2.86, *p* = 0.0094). This shows that DICS with the NZPL sCSD is much more robust to phase jitter. For paired-sampled *t*-tests comparing the two methods, NZPL sCSD performed significantly better than the full sCSD in all cases, with the exception of unjittered MEG reconstruction (*t*_(9)_ = 2.31, *p* = 0.023).

**Table 1:** Results of all *t*-tests comparing AUCs with critical AUC, and comparing AUCs between sCSD types for all phase jitter FWHMs.

	EEG			MEG		
Jitter FWHM (π radians)	Full sCSD> Critical AUC	NZPL CSD> critical AUC	NZPL CSD> Full CSD	NZPL CSD> critical AUC	Full sCSD> Critical AUC	NZPL CSD> Full CSD
0	*t*_(9)_ = −55.70,	*t*_(9)_ = 65.59,	*t*_(9)_ = 101.78,	*t*_(9)_ = 17.74,	*t*_(9)_ = 2535.28,	*t*_(9)_ = 2.31,
	n.s.	p<10^−12^	p<10^−14^	p<10^−7^	p<10^−27^	p = 0.023
0.03125	*t*_(9)_ = −54.15,	*t*_(9)_ = 162.10,	*t*_(9)_ = 105.54,	*t*_(9)_ = −7.52,	*t*_(9)_ = 2994.72,	*t*_(9)_ = 30.93,
	n.s.	p<10^−16^	p<10^−14^	n.s.	p<10^−27^	p<10^−10^
0.0625	*t*_(9)_ = −132.45,	*t*_(9)_ = 311.74,	*t*_(9)_ = 242.35,	*t*_(9)_ = −28.92,	*t*_(9)_ = 2195.15,	*t*_(9)_ = 83.63,
	n.s.	p<10^−19^	p<10^−18^	n.s.	p<10^−26^	p<10^−13^
0.125	*t*_(9)_ = −137.70,	*t*_(9)_ = 88.20,	*t*_(9)_ = 124.89,	*t*_(9)_ = −18.00,	*t*_(9)_ = 3350.15,	*t*_(9)_ = 54.02,
	n.s.	p<10^−14^	p<10^−15^	n.s.	p<10^−28^	p<10^−12^
0.25	*t*_(9)_ = −121.67,	*t*_(9)_ = 83.23,	*t*_(9)_ = 120.49,	*t*_(9)_ = −38.38,	*t*_(9)_ = 1484.72,	*t*_(9)_ = 113.44,
	n.s.	p<10^−13^	p<10^−15^	n.s.	p<10^−25^	p<10^−15^
0.5	*t*_(9)_ = −210.40,	*t*_(9)_ = 10.01,	*t*_(9)_ = 30.84,	*t*_(9)_ = −40.73,	*t*_(9)_ = 497.42,	*t*_(9)_ = 123.77,
	n.s.	p<10^−5^	p<10^−10^	n.s.	p<10^−20^	p<10^−15^
1	*t*_(9)_ = −126.51,	*t*_(9)_ = −5.96,	*t*_(9)_ = 7.52,	*t*_(9)_ = −49.22,	*t*_(9)_ = 2.86,	*t*_(9)_ = 7.59,
	n.s.	n.s.	p<10^−4^	n.s.	p = 0.0094	p<10^−4^

### Effects of Phase Lag

#### Method

To understand the effects of phase lag, simulations were repeated with the mean of the phase lag distribution varied. Phase lags of 0, 0.0625π, 0.125π, 0.25π, 0.5π, π, 1.5π and 2π radians were tested. Phase jitter and regularisation were fixed at FWHM = π/4 and *α* = 10^−6^, respectively.

#### Results

**Figure 6:**
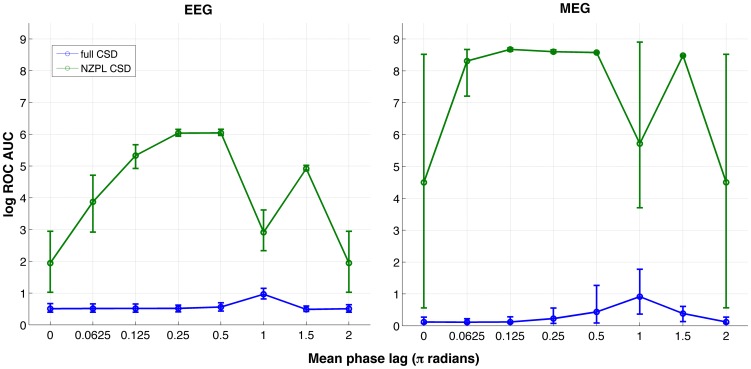
Log ROC AUCs calculated for noise-normalised coherence for EEG and MEG reconstructions, across phase lags.

AUCs for all phase lags are shown in figure 6 and *t*-test results are summarised in table 2. AUCs for all reconstructions with the full sCSD were not significantly higher than the critical AUC. For the NZPL CSD all non-instantaneous phase lags were significantly above the critical AUC (although the AUC for 0.0625π in EEG was borderline). In EEG all instantaneous phase lags (0, π and 2π) were non significant and in MEG all instantaneous phase lags were borderline significant. 1-way ANOVAs showed there was a significant effect of phase lag in EEG (*F*_(7,72)_ = 134.41, *p*<10^−38^) and MEG (*F*_(7,72)_ = 14.32, *p*<10^−11^). The effect is much stronger in EEG than MEG. Testing for differences in between the methods all showed a significant increase in AUC for the NZPL method at all phase lags in both EEG and MEG (all significant at *p*<10^−4^).

**Table 2:** Results of all *t*-tests comparing AUCs with critical AUC, and comparing AUCs between sCSD types for all phase lags.

	EEG			MEG		
Phase lag (π radians)	Full sCSD> Critical AUC	NZPL CSD> critical AUC	NZPL CSD> Full CSD	NZPL CSD> critical AUC	Full sCSD> Critical AUC	NZPL CSD> Full CSD
0	*t*_(9)_ = −113.25,	*t*_(9)_ = −5.89,	*t*_(9)_ = 6.50,	*t*_(9)_ = −149.70,	*t*_(9)_ = 2.76,	*t*_(9)_ = 7.30,
	n.s.	n.s.	*p*<10^−4^	n.s.	*p* = 0.011	*p*<10^−4^
0.0625	*t*_(9)_ = −118.36,	*t*_(9)_ = 2.71,	*t*_(9)_ = 17.05,	*t*_(9)_ = −186.32,	*t*_(9)_ = 33.5,	*t*_(9)_ = 55.32,
	n.s.	*p* = 0.012	*p*<10^−7^	n.s.	*p*<10^−10^	*p*<10^−12^
0.125	*t*_(9)_ = −121.51,	*t*_(9)_ = 23.36,	*t*_(9)_ = 55.70,	*t*_(9)_ = −167.98,	*t*_(9)_ = 595.81,	*t*_(9)_ = 521.39,
	n.s.	*p*<10^−8^	*p*<10^−12^	n.s.	*p*<10^−21^	*p*<10^−21^
0.25	*t*_(9)_ = −144.07,	*t*_(9)_ = 100.00,	*t*_(9)_ = 183.74,	*t*_(9)_ = −65.67,	*t*_(9)_ = 502.80,	*t*_(9)_ = 172.20,
	n.s.	*p*<10^−14^	*p*<10^−16^	n.s.	*p*<10^−20^	*p*<10^−16^
0.5	*t*_(9)_ = −106.39,	*t*_(9)_ = 142.72,	*t*_(9)_ = 198.92,	*t*_(9)_ = −22.55,	*t*_(9)_ = 909.55,	*t*_(9)_ = 63.04,
	n.s.	*p*<10^−15^	*p*<10^−17^	n.s.	*p*<10^−3^	*p*<10^−12^
1	*t*_(9)_ = −81.51,	*t*_(9)_ = −3.37,	*t*_(9)_ = 16.64,	*t*_(9)_ = −19.53,	*t*_(9)_ = 4.43,	*t*_(9)_ = 7.80,
	n.s.	n.s.	*p*<10^−7^	n.s.	*p* = 0.0008	*p*<10^−4^
1.5	*t*_(9)_ = −152.60,	*t*_(9)_ = 103.81,	*t*_(9)_ = 174.85,	*t*_(9)_ = −55.59,	*t*_(9)_ = 1076.97,	*t*_(9)_ = 156.91,
	n.s.	*p*<10^−14^	*p*<10^−16^	n.s.	*p*<10^−23^	*p*<10^−16^
2	*t*_(9)_ = −124.59,	*t*_(9)_ = −5.89,	*t*_(9)_ = 6.56,	*t*_(9)_ = −150.05,	*t*_(9)_ = 2.76,	*t*_(9)_ = 7.31,
	n.s.	n.s.	*p*<10^−4^	n.s.	*p* = 0.011	*p*<10^−4^

### Quantifying Phase Lag Tolerance

#### Method

A more detailed quantification of the expected phase lag tolerance of the NZPL method was obtained across a range of SNPs. Given that the full coherence between two signals with no noise is equal to 1, the coherences obtained from these simulations are a measure of the level of coherence that is retained as phase lag reduces, which we use as a metric for phase lag tolerance. Two sinusoidal waves with the same amplitude and frequency were generated, with the same parameters described previously. SNP and phase lag were systematically manipulated across different simulations: SNP was varied between 0 (all noise, no signal) and 1 (all signal, no noise); phase lags were varied between 0 and 0.5π radians. Coherence (equation 9) was calculated from the NZPL sCSD (equation 17) for each SNP and phase lag. For comparison, the imaginary coherency was also computed between the two signals.

#### Results

**Figure 7:**
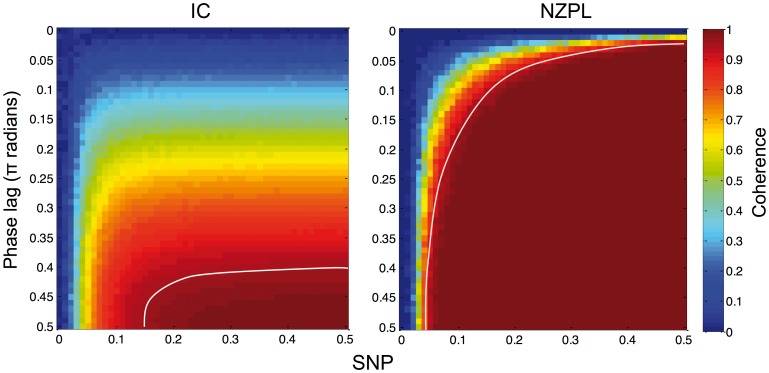
Estimated coherence calculated from imaginary coherency and NZPL coherence for a pair of sources with varying phase lag and SNR. White lines indicates estimated thresholds for coherences of 0.9.

Figure 7 shows the coherences estimated with NZPL sCSD across variation in phase lags and SNP. NZPL is clearly quite tolerant of small phase lags and low SNPs. Applying a suitable threshold to these coherence values allows the identification of minimum acceptable SNPs and phase lags. For example, the white line in figure 7 delineates the region where the simulated activity generates coherences of at least 0.9. This region encloses most of the available space indicating that the method performs well over a wide range of values. This contrasts with the coherence estimates using imaginary coherency, which is much less tolerant of variation in phase lag and SNP.

### Effects of Regularisation Parameter

DICS is sensitive to regularisation: An optimal regularisation parameter should be chosen based on the expected spatial distribution of sources. Lower regularisation increases the sensitivity of the beamformer but also is more likely to result in false positives, whereas a higher regularisation decreases sensitivity, while reducing false positives [10]. The reduction of VC/MFS artefacts in NZPL sCSD has been shown to improve the localisation of the coherent network and reduce the number of false positives in reconstructed source-level networks that would ordinarily arise in standard DICS. By eliminating these confounding factors prior to the spatial filter calculation, the dependency of reconstruction accuracy on regularisation should be less pronounced. Here, the performance of the sCSD types was tested on a range of regularisation parameters with the expectation that the NZPL method will be less sensitive to the exact value of the parameters.

#### Method

The simulations were repeated with phase lag fixed at Δ*φ* = π/2 and jitter FWHM fixed at π/4 radian and the regularisation parameter was varied using α-values between 0 and log_10_-8 in log-increments of log_10_0.5.

#### Results

**Figure 8:**
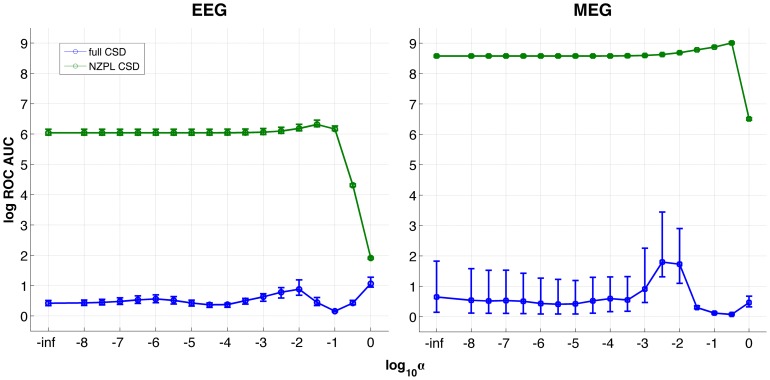
Log ROC AUCs for noise normalised coherences for EEG and MEG reconstructions, across regularisation parameters α.

Figure 8 shows the log ROC AUCs for reconstructed networks from full and NZPL sCSDs. For EEG, AUCs for full sCSD are low across much of the range with small, but still insignificant peaks at *α* = 1 and *α* = 0.01 (log_10_0 and log_10_-2 on the *x*-axis). Full CSD for MEG showed a similar pattern with a small but insignificant peak at α = log_10_2.5 with a mean AUC of 5.01. The AUCs for NZPL sCSD are uniformly high and significantly above the critical AUC for EEG (all *p*<10^−14^) and MEG (all *p*<10^−23^) with the exception of *α*≥0.1, where the AUCs shows a sudden drop off.

### Application to Human Data

To demonstrate the applicability of the NZPL modification to DICS in real human EEG recordings, the method was applied to identify coherent network components that underpin face recognition during the primary visual response that takes place in the first 400 ms of visual face presentation.

#### Method

128-channel BioSemi EEG Data for visual presentation of faces and scrambled images in a single subject [56] were obtained from the SPM website (downloaded from http://www.fil.ion.ucl.ac.uk/spm/data/mmfaces/). The paradigm consisted of 85 randomised trials of 500 ms fixation cross, followed by 600 ms presentation of either a face or a scrambled image [57]. The pre-processing was carried out using FieldTrip [54]. Data was epoched, band pass filtered at 2-45 Hz, baseline corrected and de-trended. Source reconstruction was carried out using the same procedure described for the simulations. NZPL sCSDs were calculated in a 0–400 ms, theta band (4–8 Hz) time-frequency Hanning-window for both conditions and averaged across all epochs. The BEM model and lead fields were computed from a subject-specific anatomical T1-weighted MRI using the same procedure to generate the head model for the EEG simulations. DICS was performed using this lead field and the epoch-averaged NZPL sCSD to obtain the source-level rCSDs. The coherence between all source pairs was then calculated. To obtain contrasts, analogous noise coherences were estimated using the same procedure applied to a pre-stimulus period of equal length. The true and noise coherences were Fisher transformed and then contrasted.

#### Results

**Figure 9:**
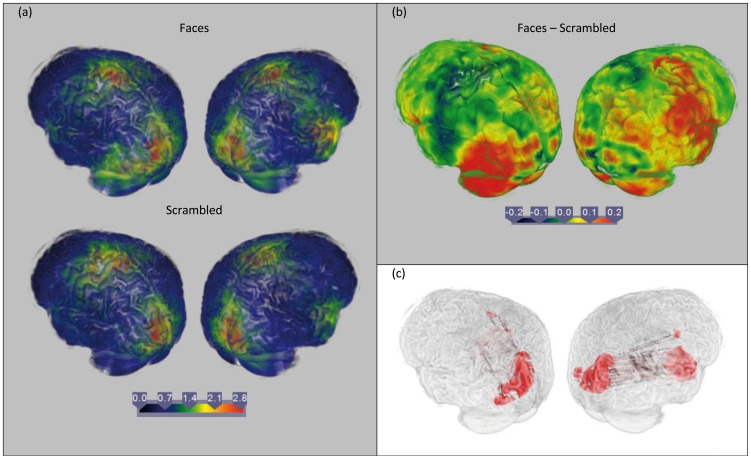
Source Coherence estimates from human EEG using DICS with NZPL sCSD. (a) shows the maximum value for each voxel value in the noise-normalised coherence matrix for faces (top) and scrambled (bottom) images at 0–400 ms, 4–8 Hz. (b) shows the maximum of the contrast between faces and scrambled connectivity matrices. (c) shows the top 0.01% of the face-scrambled contrast matrix.

Results are shown in figure 9. The highest noise contrasted coherences are plotted to visualise the most highly coherent regions. The results show that the strongest coherences in both conditions take place in primary visual cortex and the right superior temporal gyrus and precentral gyrus (figure 9a). Contrasts between the two conditions (figure 9b) show faces elicit higher coherences in the left lateralised occipital and temporo-occipital cortex and the right superior temporal region. The most strongly connected source pairs (the highest 0.01% of the connectivity matrix) within this contrast are those between the primary visual cortex and right superior temporal gyrus (figure 9c). This suggests that perceiving faces engages a direct coherent interaction between the primary visual cortex and the superior temporal gyrus. This region has been implicated in face recognition and more specifically in detecting gaze and emotion propensity [58]–[60].

## Discussion

This study aimed to optimise the dynamic imaging of coherent sources [2] method to reconstruct only non-zero phase-lagged (NZPL) interactions using a variation of imaginary coherency [1]. This approach reduces the impact of spurious interactions arising due to volume conduction (VC) and magnetic field spread (MFS) on the reconstructed source networks. To generate an unbiased symmetrical estimate of the sensor cross-spectral density (sCSD) that only embodies phase-lagged interactions, the diagonal of the imaginary part of the sCSD matrix was replaced with an estimate of NZPL-only components of the power, using eigenvector decomposition of the imaginary sCSD. This CSD is an approximation of the phase-lagged interactions with reduced phase lag bias. Using the NZPL sCSD as an approximation of the projected NZPL source interactions, an NZPL-optimised spatial filter was constructed. The projection of the NZPL sCSD using this filter significantly reduces the confounding effects of VC/MFS on source localisation are reduced. The application of the NZPL sCSD to the filter calculation is advantageous as it deliberately overestimates the signal arising from phase-lagged interactions while suppressing weaker interactions. This improves the spatial acuity of the filter. This method offers significant improvement compared to using the full sCSD for both EEG and MEG. NZPL significantly improves accuracy of the source reconstruction compared to using the full sCSD. This is shown consistently for a range of non-instantaneous phase lags and noise levels (in the form of phase jitter) and is true for both MEG and EEG data. In the case of phase jitter, using DICS with the full CSD is highly intolerant of phase jitter, unlike DICS with the NZPL CSD, which shows tolerance to even very wide distributions of phase jitter (up to FWHM = π/2). The intolerance of standard coherence measures to phase jitter has been reported previously [61].

In the case of instantaneous phase lag, the accuracy is much more inconsistent in NZPL, which is to be expected, although in the case of MEG at least, the accuracy is still good. The presence of phase jitter in these simulations means that the coherent activity can still be detected, but with much more variable accuracy. The small variances of AUCs across repetitions for NZPL compared with the full sCSD (figures 5,6 and 8) also suggest the NZPL method is more robust to noise as there is more consistency in the responses across noise-varied trials. This is consistent with the principle of imaginary coherency where the imaginary component of coherency is reduced with increasing noise [1]. Noise is attenuated by the loss of the real components and hence will not be modelled in the spatial filter or the subsequent rCSD. However, the amplitude of the phase-lagged interactions will be reduced. This may introduce scaling issues if making contrasts between networks with different levels of noise [45].

The study has also shown that the NZPL sCSD performance is more invariant to regularisation compared to the full sCSD. Regularisation reduces the sensitivity of DICS to false positives, but also increases the probability of false negatives. Higher regularisation comes at the expense of lower spatial acuity. The removal of spurious interactions by the NZPL manipulation before computation of the filter reduces the need for regularisation. NZPL therefore allows the use of spatial filters with the highest possible spatial acuity (i.e. where regularisation is set to, or close to zero).

An interesting point to note is that across all experiments the performance of the NZPL sCSD in EEG reaches a maximum AUC of about 6, while for MEG this was about 9. In addition, EEG was slightly less tolerant of small phase lags and high degrees of jitter than MEG. This is to be expected given the greater spatial acuity MEG offers in comparison to EEG and the fact that in MEG, there is less distortion of the magnetic field compared to the smearing of electrical potentials in EEG. In our simulations, this is not an issue as the NZPL method was able to reconstruct EEG networks with a high degree of accuracy. However, there may be conditions where the spatial distribution of the sensor data is too smooth to permit sufficiently accurate reconstruction. A quantification of the data smoothness such as the condition number [11] may be used as a criterion for the feasibility of source reconstruction in such cases.

In addition to tests on simulated data sets, the NZPL DICS analysis was tested using human data that compares the identification of faces with scrambled images. This appeared to elicit increased coherence between primary visual cortex and the right superior temporal cortex in the first 400 ms. The superior temporal cortex has previously been implicated in analysis of facial features, which is prominently right-lateralised [60], [62], suggesting this is a plausible subcomponent of a face recognition network. Other parts of the network may be uncovered by examining other time windows and frequency bands.

An attractive aspect of imaginary coherency is that it offers a model-free method of reducing VC/MFS artefacts [1]. This feature emerges from the fact that spurious VC/MFS interactions will always have zero phase lag. The contribution of these interactions can therefore be reduced by considering only the imaginary components. We anticipate that this attractive feature, when applied to DICS, will help to prevent the reconstruction of artefactual interactions in source space that can arise from inaccurate VC models, or from sources of electromagnetic interference external to the brain. An issue that remains however is that of higher-order VC artefacts. In any mixing of sources where there are non-zero phase lags, there will be artefactual phase-lagged coherences, both between true sources and VC artefacts, and between different artefacts. In the data presented here, this problem was not observed to any great extent, so it can be reasonably assumed that higher order artefacts are sufficiently small in NZPL as to not give rise to any false positives when reconstructing the source network. However, for more complex networks it may be more of a problem. This issue of mixing phase-lagged signals has been previously raised by Lachaux *et al* [63] in response to the assumption that VC/MFS coherences have no phase lag. In the data from the simulations presented here a small effect can be seen in the simulated sensor data (appendix S3, figure 2). The EEG full coherence plot shows first-order VC artefacts between the occipital bilateral electrodes to frontal bilateral (both contralateral and ipsilateral) electrodes. This is due to the source activity conducting to opposite sides of the head. In the NZPL coherence plot, higher order-artefacts can be seen, where occipital bilateral electrodes are weakly coherent with ipsilateral frontal electrodes, but not contralateral electrodes. The contralateral 1st order artefacts were removed by NZPL while the ipsilateral 2nd order artefacts remain. This is the same effect illustrated graphically in figure 1.

The issue described above is fundamentally the same as the EEG/MEG inverse problem, which the spatial filter resolves. The artefacts not eliminated by NZPL are still attenuated by the spatial filter. It is therefore reasonable to conclude that while the NZPL manipulation eradicates first order artefacts, higher order artefacts will remain and are still dependant on the performance of the spatial filter, and hence the accuracy of the forward model. The minimisation constraint (equation 3) will resolve this issue in the same way it does in standard DICS analysis. Additionally, the relaxation of the suppression of signals for phase-lagged sources increases the apparent SNR of the sCSD, improving its spatial accuracy (see also appendix S2). Higher-order artefacts are substantially smaller in magnitude than first-order artefacts (see appendix S3, figure 2b). However, the importance of the accuracy or complexity of the VC model to the calculation of an accurate spatial filter in the presence of higher-order artefacts remains a question. Further investigation is required to quantify this relationship.

Of course beamformers are not the only reconstruction method that can be used to explore source level connectivity and coherence is not the only functional connectivity measure that could be employed. Any functional connectivity measure can theoretically be applied to reconstructed sources to infer functional networks in source space. For instance source time-series could be estimated using the ‘virtual electrode’ method, and this could then be used to calculate coherence, synchronisation, Granger causality or transfer entropy. However, making deterministic connectivity inferences between reconstructed sources should be done with caution, as there is uncertainty about the accuracy of these reconstructions. Each reconstruction method carries with it a set of assumptions, which will give rise to some systematic error, which can contaminate connectivity estimates [12]. In particular, VC/MFS artefacts if not accounted for in the spatial filter will lead to mislocalisations of sources [29], [32]. There is also the issue of increase computational demands of separate source time-series reconstruction and coherence estimates and the issue of VC/MFS artefacts. DICS overcomes these issues as source connectivity estimates are made in a single step without the intermediary step of reconstructing the time-series. However, a particular point of concern that has arisen in connection with DICS (and any other connectivity analysis based on beamformer data) is that the covariance minimisation constraint on the spatial filter appears contradictory to the aim of identifying coherence in the source activity. The consequence of this is that reconstructed coherences are likely to be attenuated by the spatial filter. Hipp *et al* [25] regard this as an advantage because the false positive rate for reconstructed coherence is markedly reduced. This is apparent in this study by the absence of the higher-order artefact from the source reconstruction using NZPL shown in figure 3 (also see appendix S3, figure 2). It is certainly true that in the cases reported here, the covariance minimisation has not prevented the reliable detection of coherent sources, both from simulated and human data. The over-estimation of phase-lagged interaction on the NZPL CSD, compared to the full CSD, resulted in their preservation when projected to source space.

As previously discussed, the inference of connectivity in the current study depends on there being some phase lag between sources. Based on previous experimental evidence [39], it is assumed that true neural interactions would never have instantaneous phase. Given that the improvement in performance is maintained even at very small phase lags tested, Δ*φ* = 0.0625π it seems likely that this assumption will only rarely be violated. Indeed, the data in figure 7 demonstrates the robustness of NZPL to even smaller phase lags. Treating these coherence values as a metric for retention of the true coherence by NZPL for decreasing SNPs and phase lags, it can be shown that even at SNP = 0.5 (equal signal and noise), NZPL will tolerate (with 90% of coherence retained) a phase difference as small as Δ*φ* = 0.025π (approx. 4.5°). This feature addresses an issue previously raised with imaginary coherency, which is the sensitivity to the size of the phase lag. One other method shown to overcome this drawback is the phase lag index (PLI) [46], which measures the asymmetry of the distribution of phase lags. The method presented here offers a similar advantage over the original imaginary coherency method. We have shown the bias in variability of the imaginary CSD arises from the size of the phase lag (appendix S1). Removal of this bias by eigenvector decomposition therefore results in CSD estimates that remain consistent across a range of phase lags. Only when SNR is low is there an increased bias from the size of the phase lag on the inferred coherence. The reduction in SNR manifests in the case where the phase lag distribution is centred around 0 or π radians. The absence of an imaginary component at the peak of the distribution means the spatial filter has to rely on the signal obtained from the much weaker imaginary components at the periphery of the distribution. The effect is also seen in the EEG reconstruction where small phase lags are tolerated less than for MEG. However, as only two sources were simulated, we are making a generalisation from the two-source case to one with multiple sources, which may be less tolerant on small phase lags due to increased mixing between multiple sources. Further study is required to quantify the effect of number of interacting sources on phase lag tolerance.

In addition to the tolerance to small phase lags, averaging the sCSD over sufficient event-related epochs will ensure functionally relevant phase-lagged interactions are retained. This raises an issue about assumptions made concerning how neural populations interact. As noted previously, one assumption is that the probabilistic nature of encoding within stochastic firing patterns means that it is unlikely that two functionally connected neural populations are ever perfectly in phase within a given epoch. By averaging sCSDs across epochs, a different assumption is made, based on the same premise: that there is sufficient phase lag variability between epochs to render the chance occurrence of zero-lagged coherences trivial. Some other functional connectivity methods, such as phase locking value (PLV) [63], which treat phase lag consistency as a measure of functional connectivity will not detect these type of interactions. Fortunately, NZPL sCSD averaged across epochs will be sensitive to both types of interaction, with only one exception - when there is exactly zero phase lag with very low phase lag variability. It is assumed this type of interaction is sufficiently rare as to not significantly increase the false negative rate of the NZPL method.

In conclusion, NZPL DICS offers a method of significantly improving localisation of coherent networks. The method is also less computationally demanding than separately reconstructing source time-series and inferring functional networks, making it feasible to compute and perform statistical inferences on whole brain networks. This modification allows DICS to be a much more accurate tool for inferring functional connectivity from EEG and MEG recordings.

## Supporting Information

Appendix S1 **Bias in the existing imaginary coherency approach.**
(PDF)

Appendix S2 **Properties of the spatial filter.**
(PDF)

Appendix S3 **Surface-level coherence.**
(PDF)
