# Supplementary material for: Reconstructing Coherent Networks from Electroencephalography and Magnetoencephalography with Reduced Contamination from Volume Conduction or Magnetic Field Spread
Source: PLoS One. 2013 Dec 2;8(12):e81553. doi: 10.1371/journal.pone.0081553 (PMC3857849; doi:10.1371/journal.pone.0081553)
Supplement: Appendix S1 — Bias in the existing imaginary coherency approach. (PDF) [file pone.0081553.s001.pdf]

Appendix S1: Bias in the existing imaginary coherency approach

Here we highlight a source of bias in Nolte *et al*'s [1] imaginary coherency method. Since the diagonal elements of the CSD matrix (the auto-spectra) have no imaginary components, the imaginary coherency can be expressed as:

$$\mathrm{Im}\left(m_{ij}\right) = \frac{\mathrm{Im}(c_{ij})}{\sqrt{c_{ii}c_{jj}}} \text{ where } i \neq j \qquad \text{S1.1}$$

With this representation, we can show a source of bias in the imaginary coherency method. This can be demonstrated with the case where n_e=1, where there should be no variability since all the variability in the CSD should be accounted for by the variability across samples. Where n_e>1, the sensor level CSD satisfies the Cauchy–Schwarz inequality.

$$\left|c_{ij}\right| \leq \sqrt{c_{ii}c_{jj}} \qquad \text{S1.2}$$

Where n_e=1, (i.e. $c_{ij} = p_i p_j^*$) the inequality becomes the equation:

$$\left|p_i p_j^*\right| = \sqrt{p_i^2 p_j^2} \qquad \text{S1.3}$$

Meaning that all variability in the cross-spectra is accounted for by that of the auto-spectra, and vice-versa. However, following from equation 12, there is a dependence on phase lag:

$$\sin \Delta\phi_{ij} \leq 1 \qquad \text{S1.4}$$

For example, a full coherent pair of signals with a phase lag of π/4, will only have an apparent coherence of $\sqrt{1/2}$ if looking at the imaginary coherency. The consequence of this is that there are additional components in the auto-spectra that are not accounted for by the imaginary cross-spectra.

$$\mathrm{Im}\left(p_i p_j^*\right) \leq \sqrt{p_i^2 p_j^2} \qquad \text{S1.5}$$

While an exact solution to this problem is impossible (due to attenuation of imaginary components as $\Delta\phi_{ij}$ approaches zero), we can use the NZPL approach to estimate the auto-spectra such that the difference between the cross-spectra and product of auto-spectra is minimised.
