# Supplementary material for: Reconstructing Coherent Networks from Electroencephalography and Magnetoencephalography with Reduced Contamination from Volume Conduction or Magnetic Field Spread
Source: PLoS One. 2013 Dec 2;8(12):e81553. doi: 10.1371/journal.pone.0081553 (PMC3857849; doi:10.1371/journal.pone.0081553)
Supplement: Appendix S2 — Properties of the spatial filter. (PDF) [file pone.0081553.s002.pdf]

Appendix S2: Properties of the spatial filter

The advantage of using the NZPL CSD in the computation of the spatial filter for reconstructing coherent sources is demonstrated and compared to the standard spatial filter.

Method

A simple system of 62 sources and 62 sensors with a lead field mapping sources to sensors with a Gaussian smoothing kernel with a FWHM of 6mm (figure S2.1) was constructed. Two interacting sources (labelled A and B, at positions 21 and 42, figure S2.2) were defined with sinusoidal waves of 12Hz, a phase lag of π/2, amplitude of 1nA and duration of 1000ms. All other sources were silent, except for Gaussian noise with a mean of 0.1nA, which was added to the time series of all sources. 100 epochs were simulated.
Forward solutions and sCSD matrices were calculated over a 12±2Hz window, using both the full CSD and the NZPL CSD. Spatial filters were constructed, for each CSD type using equations 4 and 24 respectively. The full sCSD (i.e. the original DICS approach) was reconstructed as per equation 5. The NZPL rCSD was reconstructed as per equation 25. In addition to these approaches we also looked at the imaginary part of the full rCSD. This is to show the analogous source coherences reconstructed with the approach of [42–44]. For all three approaches, the source coherences were computed as per equation 9.
In addition to the reconstructed CSDs, a 'suppression profile' for reconstructions of the powers of the target sources, A and B and their cross-spectra, was derived. These profiles were obtained by projecting the rCSD back into sensor space, using the pseudo-inverse of the spatial filters. This value was then weighted by the original NZPL sCSD.

$$\mathbf{G}_{kl} = \mathbf{W}_l^{+}\tilde{\mathbf{C}}_{kl}\left(\mathbf{W}_k^{\mathrm{T}}\right)^{+} \circ \widehat{\mathbf{C}}_{\mathbf{X}} \qquad \text{S2.1}$$

The superscript $^{+}$ denotes the pseudo-inverse. G_{kl} denotes the filtered CSD contributing to the reconstruction of sources *k* and *l*. This shows how elements of the sCSD matrix contribute to the reconstruction of the selected rCSD matrix element.

Results

All figures plotted here show the absolute values. sCSDs are shown in figure S2.3. Spatial filters are shown in figure S2.4. Reconstructed rCSDs are shown in figure S2.5. Using the NZPL CSD for both filter calculation and reconstructing the rCSD shows focal sensitivity to the interacting sources compared to using the full sCSD filter. The imaginary part of the full rCSD shows better sensitivity to the true interactions, but the spatial acuity is still comparable to the full rCSD. Similar results were observed with the reconstructed coherences (figure S2.6): The full and imaginary coherency using the full filter both show poor spatial acuity compared to that derived from the NZPL filter. In terms of the reconstructed power, the NZPL filter shows a greater spatial acuity compared to the full filter (figure S2.7).
The suppression profiles (figure S2.8) show how the full and NZPL filters differ in composition. The reconstructed rCSD with the real filter derives from many elements of the sCSD. The sCSD shows roughly the same suppression profile for both power and cross-spectra. There is little preferential filtering of the cross-spectra compared to the power. For the NZPL sCSD, there is much stronger suppression of the sCSD when reconstructing source power, while there is a noticeably weakened suppression of the cross-spectra in the sCSD when reconstructing source cross-spectra.

These simulations show that using the NZPL sCSD for filter computation relaxes the suppression of coherent activity from sensor pairs that have a high phase lagged coherence. This is in contrast to the filter computed from the full sCSD, which uniformly suppresses activity from all non-target sources, including those with high phase lagged coherence with the target, which we wish to retain. The result of this is that the activity from these coherent sensor pairs will contribute more to the rCSD of the target source pair. This increases the apparent SNR of the signal, which contributes to greater spatial acuity [11]. The relaxation of the spatial filter in this way also leads to a beneficial overestimation of the cross spectra when the spatial filters from two highly coherent target sources are multiplied in equation 25.

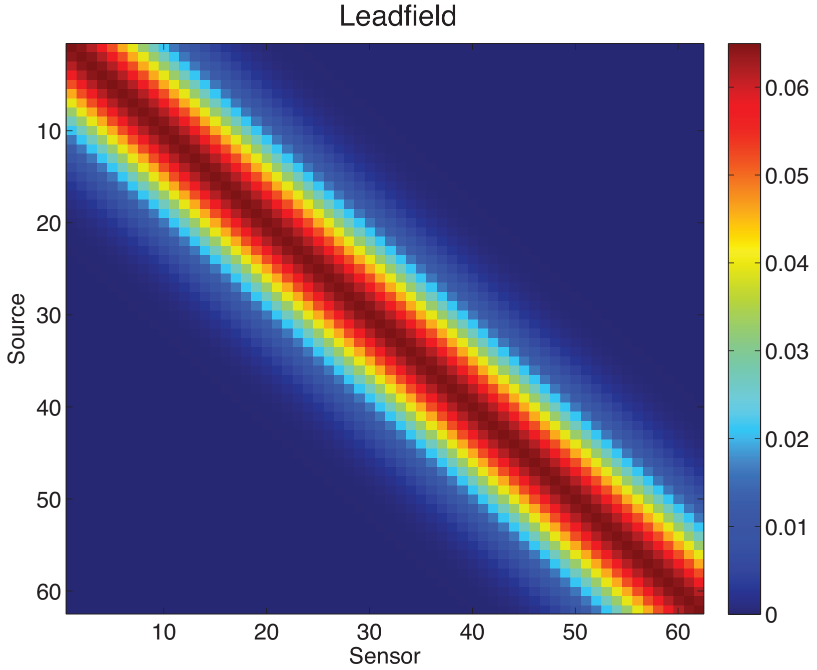

Figure S2.1. The CSD of the original source interactions. Coherent sources are placed at positions 21 and 41.

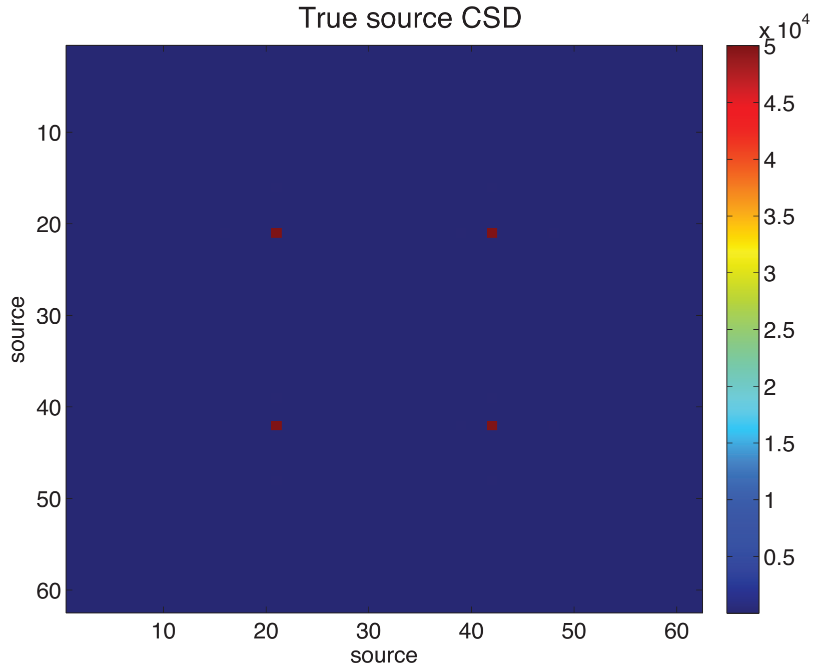

Figure S2.2. The lead field matrix used to generate forward solutions from the original CSD.

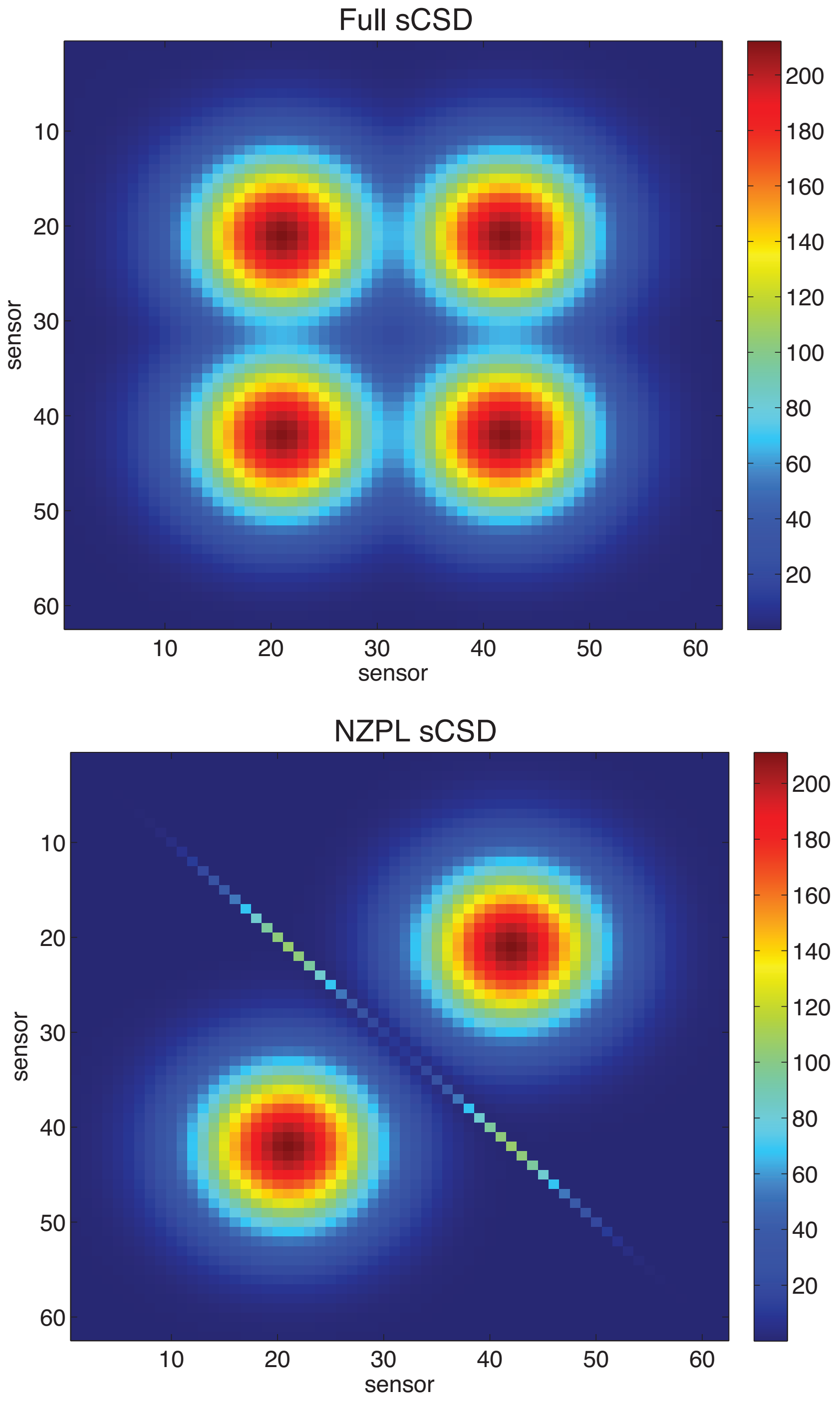

Figure S2.3. Full and NZPL sCSD matrices.

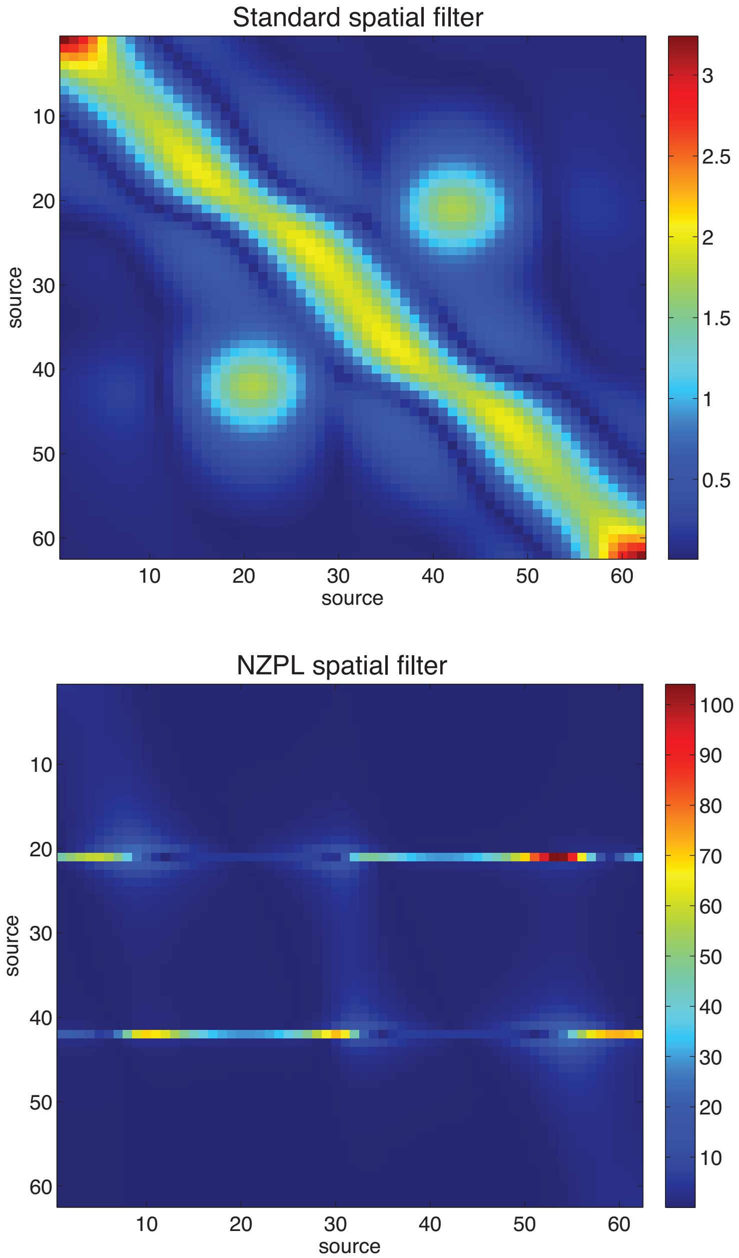

Figure S2.4. Spatial filters computed from full and NZPL sCSD matrices

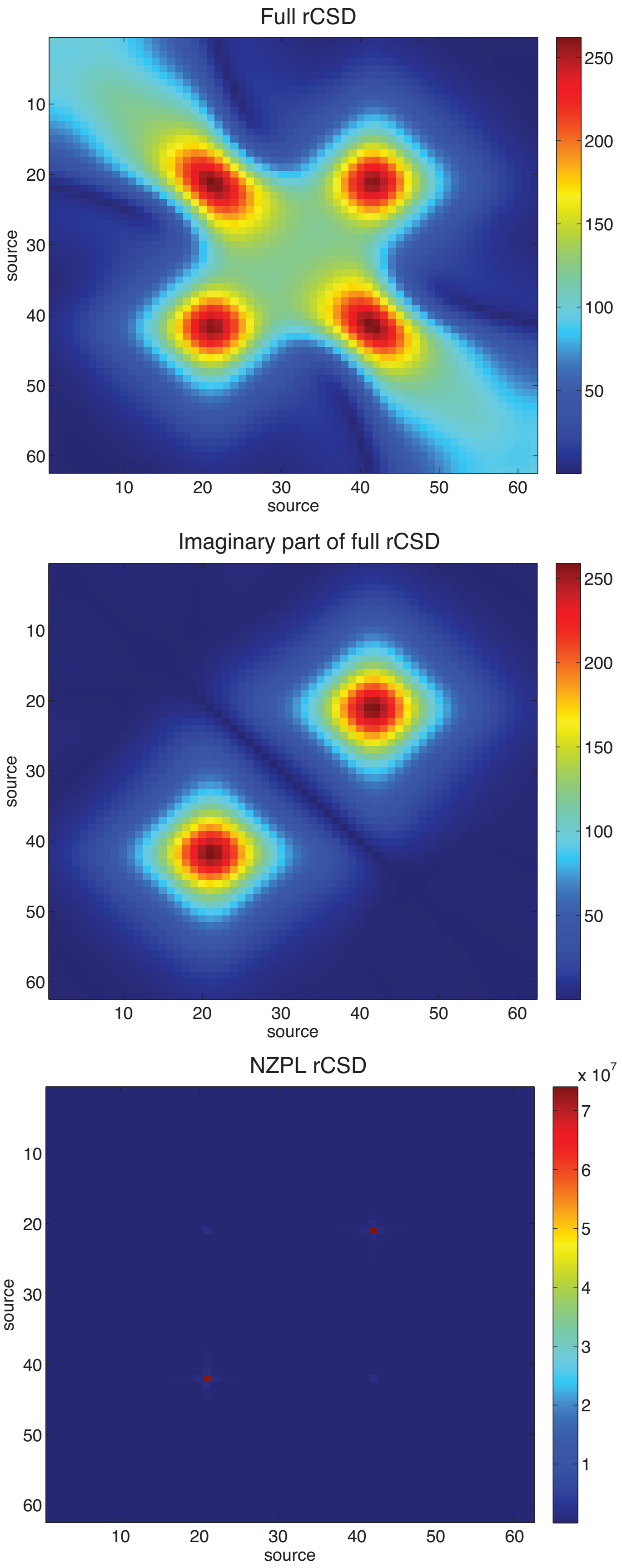

Figure 5. Full and imaginary rCSDs computed from the full sCSD with the standard filter, and NZPL rCSD computed with the NZPL filter.

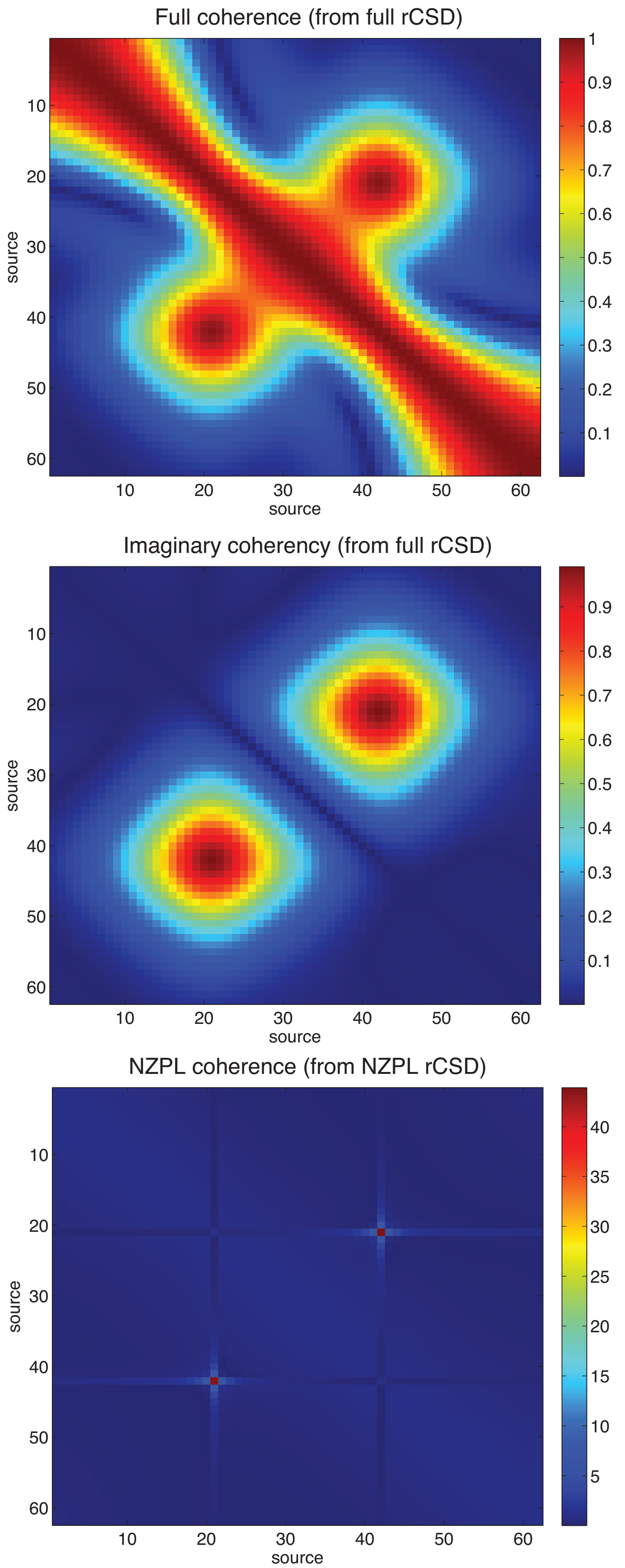

Figure S2.6. Reconstructed coherence and imaginary coherency from the full rCSD and coherence from the NZPL rCSD.

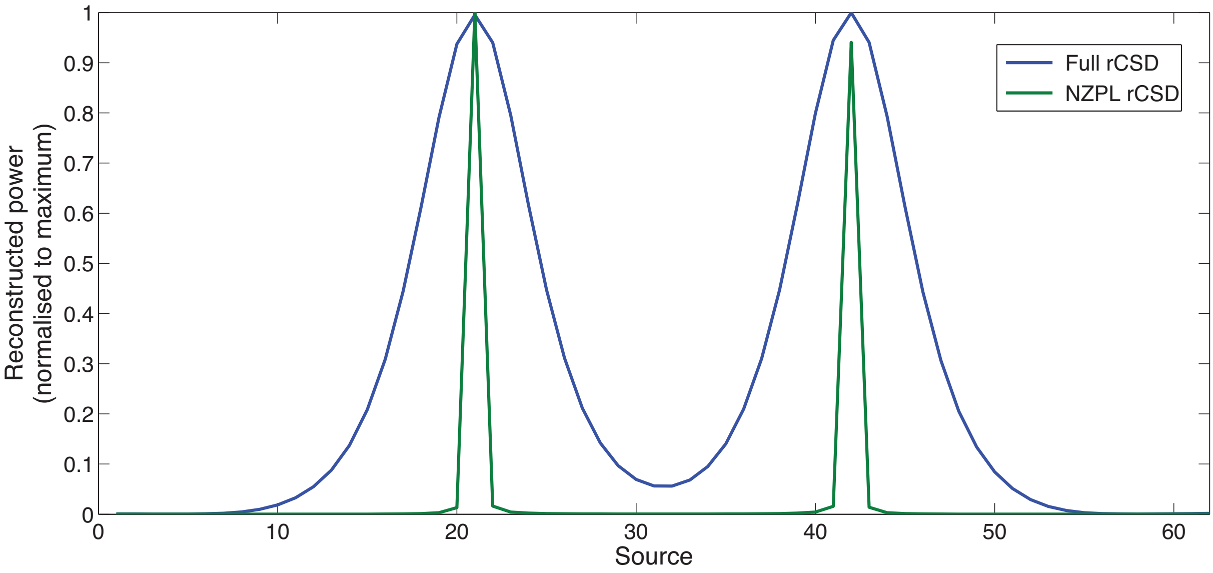

Figure S2.7. Reconstructed source power from the full and NZPL rCSDs.

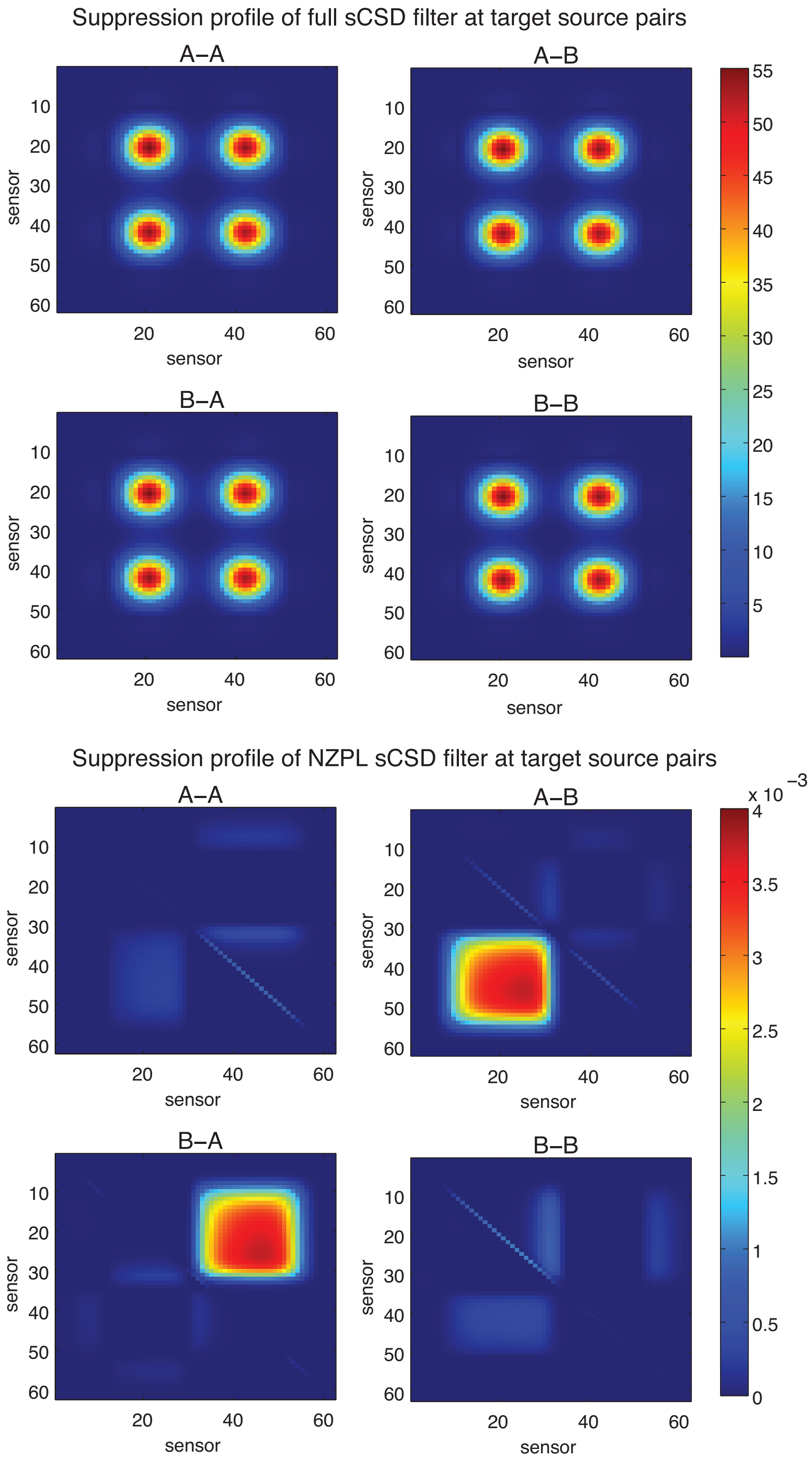

Figure S2.8. Suppression profiles of reconstructed rCSDs for sources A and B, constructed with the full filter and the NZPL filter.
