# Supplementary material for: Reconstructing Coherent Networks from Electroencephalography and Magnetoencephalography with Reduced Contamination from Volume Conduction or Magnetic Field Spread
Source: PLoS One. 2013 Dec 2;8(12):e81553. doi: 10.1371/journal.pone.0081553 (PMC3857849; doi:10.1371/journal.pone.0081553)
Supplement: Appendix S3 — Surface-level coherence. (PDF) [file pone.0081553.s003.pdf]

Appendix S3: Surface-level coherence

To demonstrate the improvements of the NZPL sCSD for examining interactions on the sensor level, coherences calculated from the sCSD were plotted.

Method

The two sCSDs were calculated for simulated data using a phase lag of $\Delta\phi=0.5\pi$. Surface-level coherences were calculated as per equation 9. The topographic distribution of the power generated by EEG and MEG lead fields is plotted along with the coherence topography for each sCSD type using the coherence visualisation method from Nolte *et al* [1]. Each topographic plot is positioned in a topographic layout for each sensor and shows the coherence from that sensor to all other sensors.

Results

The topographic plots of power within the frequency range of interest are shown in figure S3.1. The EEG topography shows the peak activity over the point nearest the sources in the bilateral occipital regions. There are also some noticeable VC artefacts in more anterior electrodes. The topography of MEG shows peak power radially around the source points, consistent with the axial orientation of the gradiometers.

The EEG coherence plots calculated from the absolute full sCSD (as is done to calculate traditional coherence) and the NZPL coherences are shown in figure S3.2. For the full CSD, there is widespread coherence between all sensors except the extreme temporal electrodes. Of particular note are the electrodes on bilateral frontal regions showing high coherence with the ipsilateral and contralateral occipital electrodes. The NZPL CSD coherences show the bilateral occipital coherences much more clearly and show the same topographic distribution as the power. In addition, the bilateral occipital electrodes show weak coherences between the ipsilateral, but not the contralateral frontal electrodes. This is attributed to higher-order artefacts that the NZPL method alone is unable to eliminate.

The coherence distribution for MEG shows a similar pattern (figure S3.3). For the full CSD, there is almost full coherence between all gradiometers except those most distal from the source. The NZPL coherences show clear coherences between the induced magnetic fields of the bilateral occipital sources.

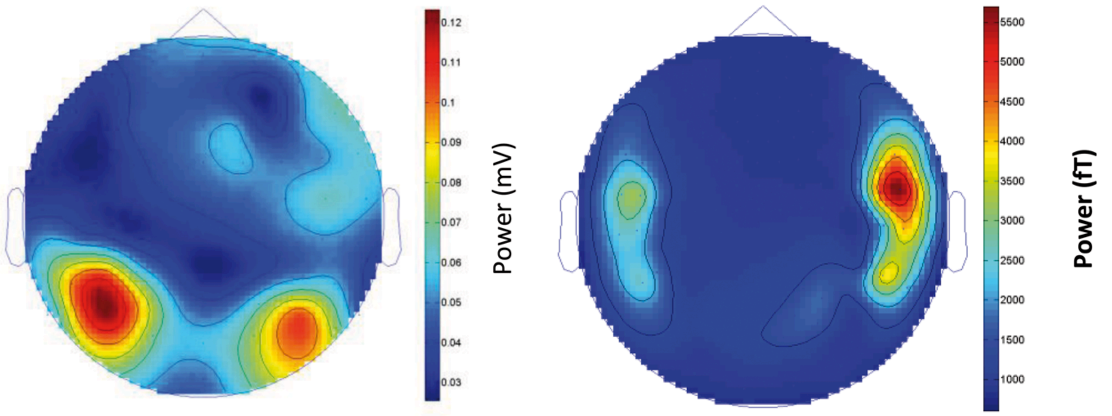

Figure S3.1. Topographic distribution of surface power for EEG and MEG within a 25-40Hz window.

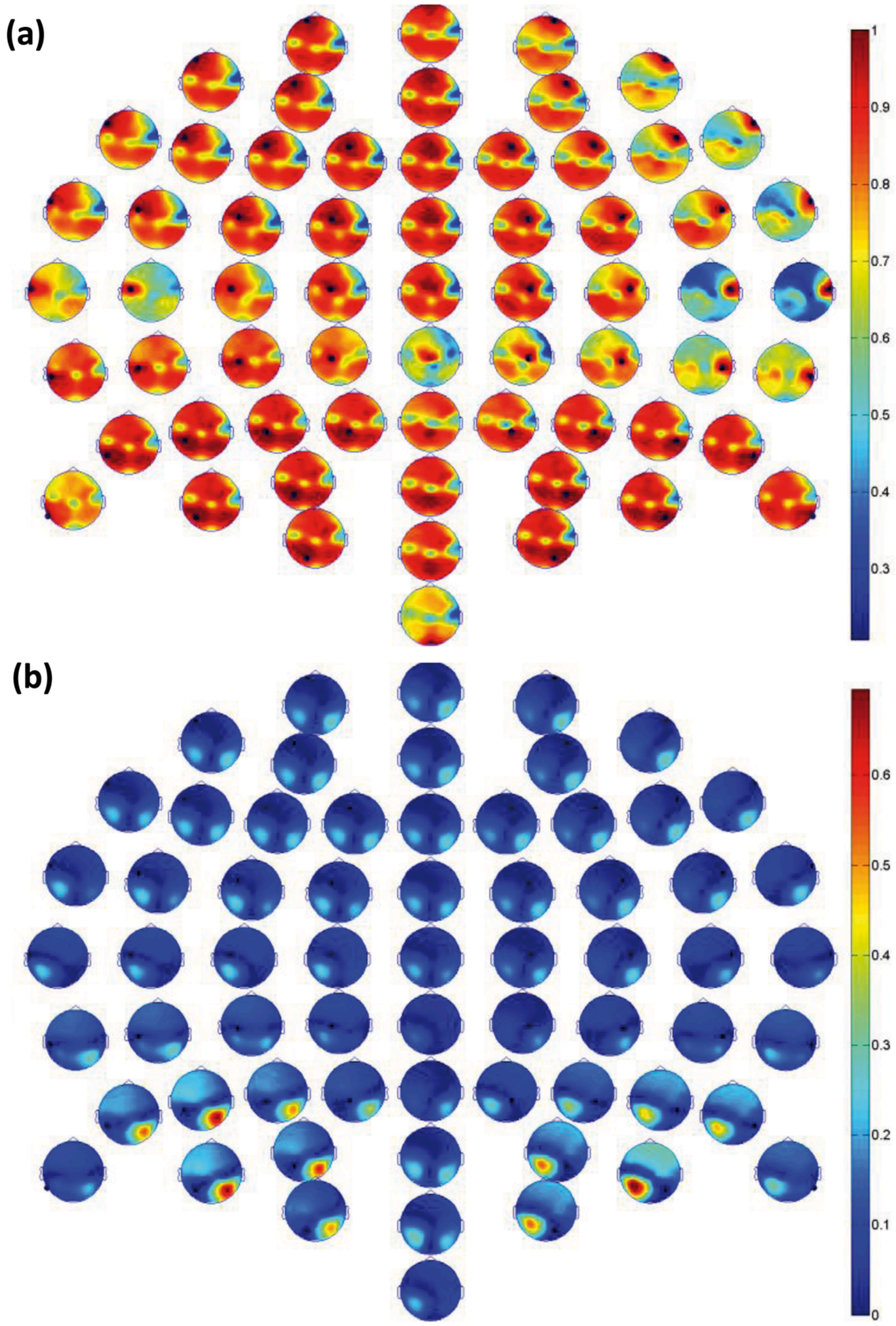

Figure S3.2. Sensor level coherence for EEG calculated using (a) full CSD and (b) NZPL sCSD for network with phase lag of $\Delta\phi=\pi/2$ Coherence is shown in a distributed series of topographic plots. Each topographic plot shows the coherence from that sensor position to all other sensors.

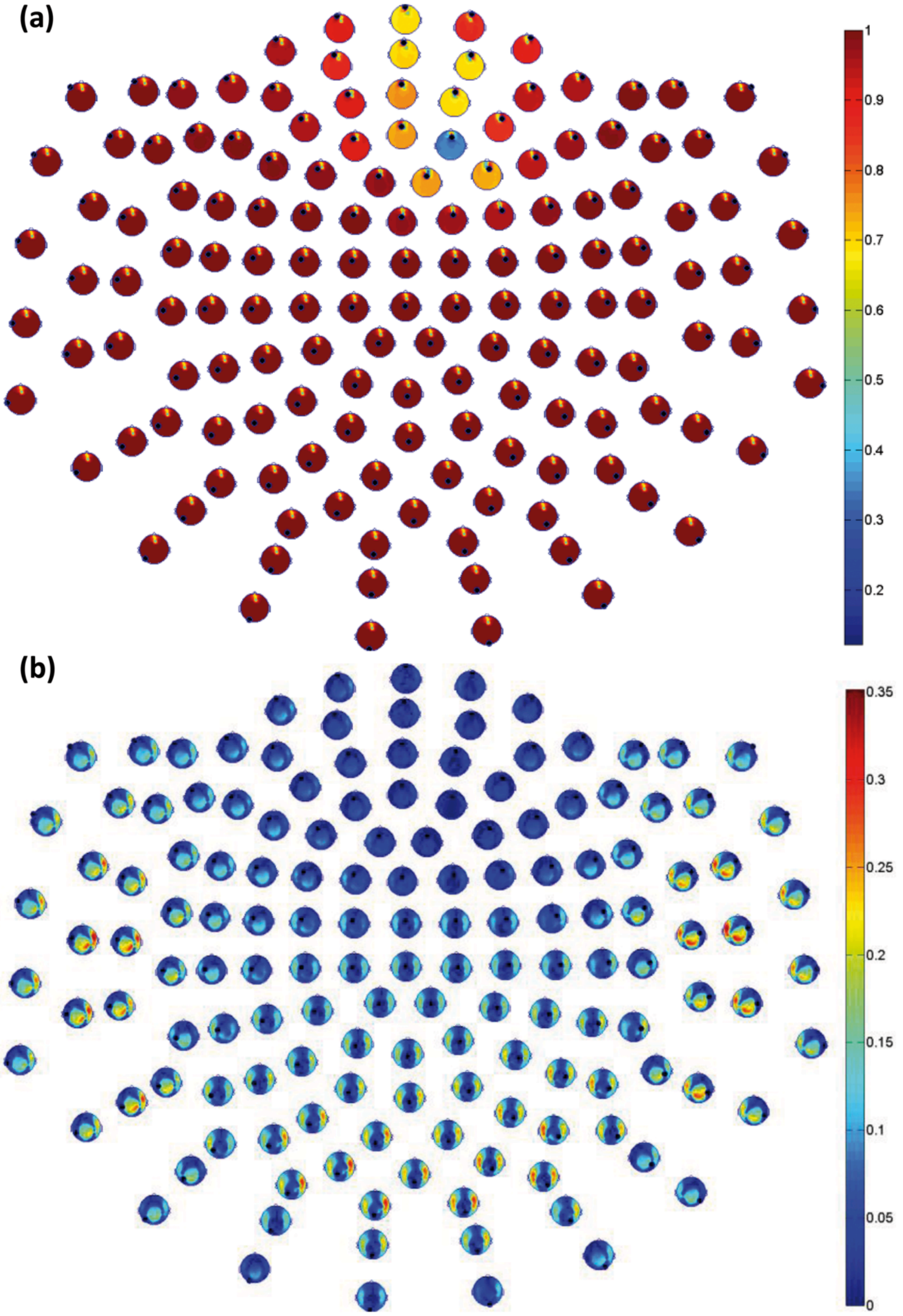

Figure S3.3. Sensor level coherence for MEG calculated using (a) full sCSD (b) NZPL sCSD for network with phase lag of $\Delta\phi=\pi/2$
